# The type I ribosome-inactivating protein α-MMC induced significant apoptosis of lung cancer A549 and 95-D cells by activating the caspase cascade through TNF signaling pathway

**DOI:** 10.3389/fphar.2025.1529151

**Published:** 2025-06-09

**Authors:** Di Yang, Di Peng, Houke Li, Di Jia, Yiping Zhou, Bintao Hu, Wei Chen, Yao Meng

**Affiliations:** ^1^ The Second Affiliated Hospital of Chengdu Medical College, China National Nuclear Corporation 416 Hospital, Chengdu, China; ^2^ School of Laboratory Medicine, Chengdu Medical College, Chengdu, China; ^3^ West China School of Public Health and West China Fourth Hospital, Sichuan University, Chengdu, China

**Keywords:** ribosome-inactivating protein, α-MMC, lung cancer, antitumor activity, apoptosis, signaling pathway

## Abstract

**Background:**

Ribosome-inactivating proteins (RIPs) are a class of toxic proteins with RNA N-glycosidase activity, primarily found in plants. Due to their antiviral, antibacterial and anti-tumor biological activities, RIPs have received extensive attention all over the world. Alpha-momorcharin (α-MMC) is a typical type I ribosomal inactivation protein, showing excellent anti-tumor activity. Lung cancer is a leading cause of global morbidity and mortality; however, current treatments remain limited, and patient prognosis is poor.

**Methods:**

In this study, α-MMC was extracted from *momordica charantia* seeds, and a series of *in vitro* studies were carried out on lung cancer A549 and 95-D cells, such as cell proliferation, cycle, apoptosis, migration to invasion, etc. Further, Western blot was used to explore the Cyclin-CDK-CKI signaling pathway, Caspase cascade and TNF signaling pathway respectively.

**Results:**

Studies have shown that α-MMC can significantly inhibit the proliferation of lung cancer A549 and 95-D cells. α- MMC can co-mediate the TNF signaling pathway to participate in cell regulation through NF-κB (down-regulated p65/p50) and MAPK (downregulated p38/JNK) signaling pathways, and activate downstream effector factors of Caspase to induce apoptosis. The expression of Cyclin D, CDK4, Cyclin A and CDK2 was downregulated by cyclin-CDK-CKI signaling pathway, thus blocking the cell cycle in G0/G1 phase or S phase.

**Conclusion:**

α-MMC exhibited significant antitumor activity against lung cancer A549 and 95-D cells, which laid the experimental foundation for clinical research and development of novel anti-tumor drugs.

## 1 Introduction

Ribosome-inactivating proteins (RIPs) are a class of toxic proteins that activate RNA *N-*glycosidase. By removing adenine at position 4,324 of the specific sequence of eukaryotic 28S rRNA, RIPs damage the structure and biological activity of ribosomes and inhibit protein biosynthesis (Barbieri et al., 1993). Existing studies have shown that RIPs have various effects, including anti-bacteria, anti-HIV, anti-tumor, promoting immune regulation and other biological activities, which has attracted wide attention in the agricultural and biomedical fields (Akkouh et al., 2015; Zhang et al., 2020; Zhu et al., 2021; Wang et al., 2023). The discovery of RIPs has a history of more than 100 years, and a variety of RIPs have been screened from more than 350 species of plants and a few fungi, algae and bacteria (Nielsen and Boston, 2001). They all exhibit biological activities that inhibit protein synthesis, but the mechanism of actions is not exactly the same (Fong et al., 2000). According to the structure of RIPs, it can be divided into three types. Type I RIPs are a class of single peptide-chain basic proteins with molecular weight of about 30kDa, which mainly exist in plants. They can fuse with cell membrane through receptor-mediated endocytosis, thus entering cells to inactivate ribosomes, and have no obvious toxic effect on normal cells (Stirpe et al., 1992; Puri et al., 2009). Type II RIPs are a class of heterodimeric acidic proteins with molecular weight of about 60 KDa. The two peptide chains A and B are connected by disulfide bonds, in which chain A has excellent biological activity, similar to type I RIPs, and chain B has specific galactoagglutinin activity, which can assist chain A to enter cells and exert RNA *N-*glycosidase activity (Endo and Tsurugi, 1987; Puri et al., 2009). Type III RIPs are relatively rare single-chain proteins, predominantly identified in maize and wheat, and there are few relevant reports.

Screening for compounds with anti-cancer activity from plant-derived natural products represents one of the crucial approaches in the research and development of novel anti-tumor drugs (Wang et al., 2024; Zhu et al., 2024). As a traditional Chinese medicine, bitter melon has been found to contain various types of RIPs, among which type I RIPs mainly include α-momordicin, β-momordicin, γ-momordicin, δ-momordicin, ε-momordicin, MAP30 and so on (Jia et al., 2017). Among them, α-MMC, as a classic type I ribosome-inactivating protein, is a relatively high content of ribosome-inactivating protein extracted from bitter melon seeds at present, showing good anti-tumor activity. Relevant studies have confirmed that α-MMC can selectively kill chorionic cancer cells, melanoma cell lines JAR and EMT-62058, sarcoma S180 cells, liver cancer cells and so on (Tsao et al., 1990; Yao et al., 2011). At the same time, Manoharan et al. further confirmed the anti-tumor activity of α-MMC and β-MMC, and found that they both had significant growth inhibition effects on human astroglioma cells 1321N1, U87-MG and malignant melanoma cells SkMel (Jiang et al., 2018). However, it has no toxic effect on human healthy muscle cells L6, and it has been proved that α-MMC and β-MMC exert anti-tumor effects by significantly enhancing the activities of caspase-3 and caspase-9 and promoting the release of cytochrome C to induce cell apoptosis (Manoharan et al., 2014). In conclusion, existing studies have shown that bitter melon RIPs have excellent anti-tumor activity and has certain clinical application value, which has been widely concerned by researchers.

Cancer has always been a major threat to human health, seriously affecting people’s physical health, and has a serious negative impact on society and economy. According to the latest Global Status report on malignancies, lung cancer has been the main malignant tumor endangering human health among all the frequent cancers (Sung et al., 2021). With 2.2 million new cases and 1.8 million deaths worldwide in 2020, lung cancer is the second most commonly diagnosed cancer and the leading cause of cancer death after heart disease, accounting for about one-tenth (11.4%) of cancers diagnosed and one-fifth (18.0%) of deaths (Sung et al., 2021). Lung cancer is the leading cause of cancer incidence and death in men. Among women, the incidence of lung cancer ranks fourth after breast cancer, cervical cancer and colorectal cancer, and the death rate ranks second after breast cancer (Cao et al., 2021). However, in clinical treatment, the main treatment of lung cancer is radiotherapy and chemotherapy, which has greater toxicity and side effects, poor prognosis and expensive treatment (Foerster et al., 2022). Therefore, the development of anti-tumor drugs has been the focus of research at home and abroad.

Under the above research background and *status quo*, we extracted α-MMC from bitter gourd and applied α-MMC to lung cancer cell lines A549 and 95-D, conducting a series of *in vitro* anti-tumor activity studies. This study aims to find a breakthrough point through transfer group sequencing, and carry out research on cell proliferation, cycle, apoptosis, migration and invasion, and gradually conduct in-depth research on apoptosis pathway, TNF signaling pathway and other mechanisms. The aim is to reveal the molecular mechanism of α-MMC’s anti-tumor activity and lay a foundation for the development of α-MMC as clinical anti-tumor drugs.

## 2 Materials and methods

### 2.1 Isolation and purification of α-MMC

According to the method of purifying RIPs reported in previous literature (Meng et al., 2014), α-MMC was successfully extracted from Momordica charantia L. Seventy grams of bitter melon seeds were ground and crushed, and stirred with 500 mL PBS (pH 6.5, 0.02 M sodium phosphate containing 0.15 M sodium chloride) for 48 h. The crude protein extract was obtained by collecting the supernatant after centrifugation. Subsequently, the target protein α-MMC was obtained by acidification, 30∼65% ammonium sulfate fractionation, cation exchange chromatography, molecular sieve chromatography and linear gradient elution (0.15 M NaCl pH6.5 0.02 M phosphate buffer). The molecular weight of α-MMC was identified by SDS-PAGE electrophoresis. The final product was filtered by 0.22 μm membrane and stored at 4°C for *in vitro* studies.

### 2.2 RNA extraction and illumina high-throughput transcriptome sequencing

A549 cells were inoculated into 6-well plates at a density of 1 × 10^5^ cells/mL, incubated overnight, and then treated with 120 ug/mL α-MMC for 72 h. At the same time, the control group without drug intervention was set up, and each group was set up for 3 replicates. After 72 h of treatment, pancreatic enzymes digested and collected the cells, and then the cells were commissioned to Beijing Tsingke Biotech Co., Ltd. For RNA extraction, library construction and RNA sequencing.

### 2.3 Cell culture

The human lung adenocarcinoma cell line A549 and the human highly metastatic lung cancer cell line 95-D were purchased from the American Type Culture Collection. The cells were incubated in RPMI-1640 (Hyclone, Inc., Logan, Utah, United States) supplemented with 10% fetal bovine serum (FBS, Hyclone, Inc., Logan, Utah, United States), penicillin (100 U/mL) and streptomycin (0.1 mg/mL). Cells were cultured at 37°C in an incubator containing 5% CO_2_ and expanded through splitting 1:3 twice a week. Cells in the exponential growth phase (∼1 × 10^6^ cells/mL) were used for the following experiments.

### 2.4 Cell proliferation assay

Following the manufacturer’s instructions, cell proliferation was determined using the Cell Counting Kit-8 (CCK-8; Biosharp Technology Co., LTD., Anhui, China). In detail, 1 × 10^4^ cells/well were seeded into 96-well plates and cultured in 100 µL RPMI-1640 medium overnight. With 0 μg/mL α-MMC as the control, A549 cells were treated with various concentrations of α-MMC (15, 30, 60, 120, 240, 480, and 960 μg/mL), and 95-D cells were treated with 5, 10, 20, 40, 80, 160, and 320 μg/mL α-MMC, respectively. For performing the CCK-8 assay, 10 µL CCK-8 reagent was added to each well and the plates were incubated at 37°C for 1 h. The cell viability was measured every 24 h, and the optical density (OD) was measured at 450 nm using a microplate reader. The data were analyzed by Graphpad 8.0.2 to calculate IC_50_, and the appropriate concentration of α-MMC was selected for subsequent experiments.

### 2.5 Cell morphology analysis

The effect of α-MMC on morphological changes of A549 and 95-D cells was analyzed by phase contrast microscopy (OLYMPUS BX63, shanghai, China). These two cell lines were inoculated into 6-well plates at a density of 1 × 10^5^ cells/mL. When the cell growth reached 50%, A549 and 95-D cells were treated with α-MMC for 48 h. The cell morphology was recorded under the microscope at ×100 magnification using three randomly selected visual fields.

### 2.6 Colony formation assay

A549 and 95-D cells were inoculated on a 6-well plate by 1,000 cells/well and cultured overnight. The cells were replaced with complete culture medium after they were treated with different concentrations of α-MMC for 24 h. Subsequently, the cells were incubated for about 14 days until a monoclonal community formed. The cells were fixed with 4% paraformaldehyde for 30 min, stained with 0.1% crystal violet for 30 min, and then photographed.

### 2.7 Flow cytometry cell cycle assay

Following the manufacturer’s protocol, cell cycle A549 and 95-D cells were evaluated using a propidium iodide (PI) Cell Cycle Detection kit (KeyGEN BioTECH, Nanjing, China). A549 cells were treated with α-MMC for 48 h while 95-D cells were treated for 24 h to detect the cell cycle. The cells were washed with cold PBS and mixed in 70% ethanol at 4°C overnight. After resuspension with dyeing working solution containing 50 µL Rnase A and 450 µL PI, cells were incubated at room temperature for 30 min and then analyzed by flow cytometer.

### 2.8 Cell migration assay

The cell migration ability was detected by wound healing experiment. 5 × 10^5^ cells were inoculated on the 6-well plate. When the cells evenly spread the hole plate, two vertical lines were drawn on the plate with 200 µL gun tip. The cells were washed three times with PBS and cultured in serum-free medium with different concentrations of α-MMC. With 0 h as the control, the scratch width at the same position was observed at different time points (0, 6, 12, 24, 48 h) under the microscope at ×40 magnification. Then the migration area was analyzed by ImageJ software.

### 2.9 Transwell invasion assay

Cell invasive ability was assessed using 24-well Transwell chambers (EMD Millipore, Billerica, MA, United States) with membrane pore size of 8.0 µm. To simulate the cell membrane, Transwell membranes were precoated with Matrigel^®^ (BD Biosciences, San Jose, CA, United States) at 37°C for 60 min prior to the experiment. In detail, 100 µL serum-free medium with 1 × 10^5^ cells was plated in the upper chambers of the Transwell plates, while 500 µL medium containing 10% FBS was added to the lower chamber. Following incubation at 37°C with 5% CO_2_ for 48 h, non-migrating cells on the top chamber were scraped off with cotton-tipped swabs, and cells that had migrated through the membrane were fixed with 4% paraformaldehyde at room temperature for 30 min. Cells were stained with 0.1% crystal violet at room temperature for 20 min. Subsequently, the migrated cells were counted under a microscope at ×100 magnification using five randomly selected visual fields.

### 2.10 Flow cytometry apoptosis assay

Apoptotic cells were evaluated using an Annexin V-fluorescein isothiocyanate (Annexin V-FITC)/propidium iodide (PI) Apoptosis Detection kit (KeyGEN BioTECH, Nanjing, China). The apoptosis rate was detected after α-MMC treated A549 cells for 72 h and 95-D cells for 48 h. The cells were harvested by trypsinization without EDTA, washed twice with cold PBS, centrifuged at 2000 rpm at 4°C for 5 min. A total of 5 µL FITC-conjugated Annexin V and 5 µL PI were added and incubated for 10 min at room temperature in the dark. Following the cells were analyzed using a flow cytometer.

### 2.11 Hoechst staining analysis

After α-MMC treatment of A549 cells for 72 h and 95-D cells for 48 h, the cells were stained with 500 µL Hoechst33342 (KeyGEN BioTECH, Nanjing, China) solution at 37°C for 20 min without light. Then cell morphology was observed under ultraviolet light stimulated by fluorescence microscopy.

### 2.12 Cellular ROS detection

Intracellular ROS levels were detected by Reactive Oxygen Species (ROS) assay kit (Nanjing Jiancheng Biotechnology, Nanjing, China). Following α-MMC treated A549 cells for 72 h and 95-D cells for 48 h, cells were collected and incubated with 10 μM DCFH-DA at 37°C for 20 min. Then the resuspending cells with 100 µL PBS were analyzed by flow cytometer.

### 2.13 Mitochondrial membrane potential (MMP) assay

Following α-MMC treated A549 cells for 72 h and 95-D cells for 48 h, the MMP of the cells was detected by JC-1 probe (Solarbio Technology Co., LTD., Beijing, China). After the cells were collected and washed with PBS, they were stained with JC-1 staining solution for 20 min away from light. Then cells were washed twice and resuspended with JC-1 dye buffer (1×). The MMP of the cells was measured by flow cytometry and inverted fluorescence microscopy, respectively.

### 2.14 Western blot analysis

Following α-MMC treated A549 cells for 72 h and 95-D cells for 48 h, total proteins were extracted from the cells using cell lysis buffer (Solarbio Technology Co., LTD., Beijing, China). Following lysis for 30 min on ice, the lysates were centrifuged at 10,000 × g at 4°C for 10 min. The Bicinchoninic Acid Protein Quantitative kit (Solarbio Technology Co., LTD., Beijing, China) was used to determine the protein concentration. A total of 20 µg protein was separated via SDS-PAGE on an 12.5% Tris-glycine gradient gel. Subsequently, the proteins were transferred onto a PVED membrane for 2 h and blocked with 5% non-fat milk in TBS containing 0.1% Tween-20 (pH 7.4) at room temperature for 2 h. The membrane was incubated in TBST for specific primary antibody (1:1,000, Proteintech Group, Inc., Chicago, United States) at 4°C overnight, and for goat anti-rabbit/mouse horseradish peroxidase-conjugated secondary antibody (1:1,000) at room temperature for 2 h. Primary and secondary antibodies were from Abcam (Proteintech Group, Inc., Chicago, United States). Western blot bands were visualized using an enhanced chemiluminescence reagent system (PTG Company; United States) and were quantified using Image Lab 6.0.1 software (Bio-Rad Laboratories, Inc., United States).

### 2.15 Statistical analysis

Each group of experiments was repeated three times and the relevant data were represented by mean value ±standard deviation. SPSS 26.0 and Graphpad 8.0.2 were used for statistical analysis. The data was performed by using one-way or two-way analysis of variance (ANOVA) and Tukey’s *post hoc* test. P < 0.05 was considered to indicate a statistically significant difference between values.

## 3 Results

### 3.1 Analysis of yield and purity of α-MMC

As shown in Supplementary Figure S1, the extracted solution retained in each purification step was identified by SDS-PAGE electrophoresis for molecular weight size. It can be seen that with the advancement of purification step, miscellaneous proteins were gradually removed and finally a single target protein was obtained. Lane 1 represents the crude enzyme extract, which contains the total proteins from bitter melon seeds. Lane 2 shows the proteins after ammonium sulfate fractionation, where the target protein is preliminarily separated from heterogeneous proteins and enriched. Following purification by a cation exchange column, the proteins in Lane 3 have eliminated large-molecular-weight impurities, with the target protein undergoing further purification. After molecular sieve separation—and as confirmed by our preliminary detection—Lane 4 contains only MAP30 and α-MMC. Through separation on a cation exchange column using a salt ion gradient, MAP30 and α-MMC are resolved, and the final Lane 5 represents the purified α-MMC. Further determination of protein concentration showed that 56.61 mg α-MMC was successfully extracted with a yield of 0.92% (Supplementary Table S1). The successful extraction of α-MMC provides sufficient raw materials for further research.

### 3.2 Transcriptome difference analysis of A549 cells induced by α-MMC

**FIGURE 1:**
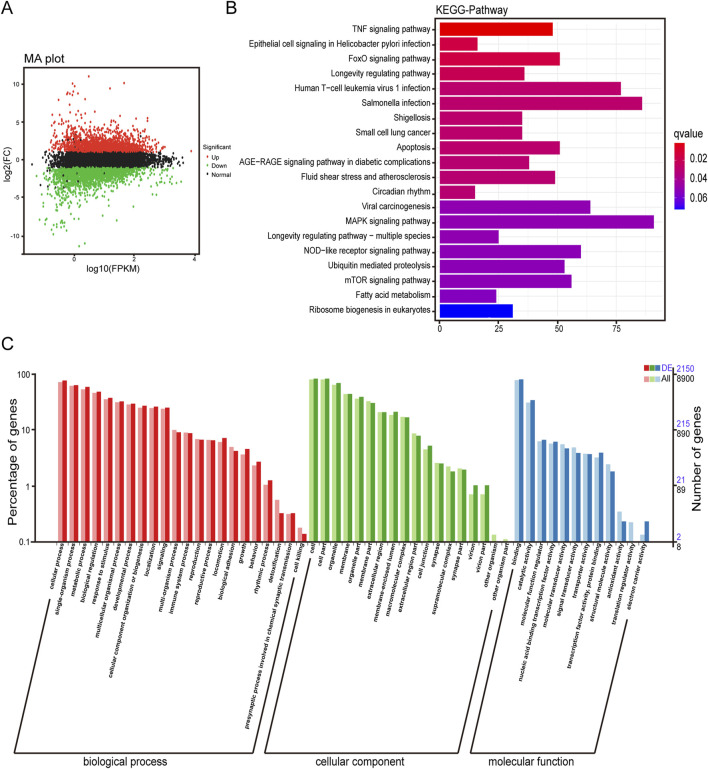
Transcriptome difference analysis of A549 cells induced by α-MMC. A549 cells were treated with 120 μg/mL α-MMC for 72 h and transcriptome differences were analyzed by high-throughput sequencing. **(A)** Differential expression volcano plot; **(B)** Differential expression KEGG classification map; **(C)** Differential expression GO classification. Note: The green/red/black dots in **(A)** represent downregulated/upregulated/non-differentially expressed genes respectively; In **(C)**, DE represents the differentially expressed gene background, and All represents the entire gene background.

In order to explore the anti-tumor molecular mechanism of α-MMC, 120 μg/mL α-MMC was treated with A549 cells for 72 h, and differential gene expression and gene function were analyzed by Illumina transcriptome sequencing. Transcriptome analysis of the volcano plot showed that there were 4,973 differentially expressed genes in the α-MMC treatment group, including 2,333 upregulated genes and 2,640 downregulated genes (Figure 1A). KEGG enrichment analysis of differentially expressed genes showed the top 20 pathways with the lowest Q value according to the enrichment significance. The results showed that differentially expressed genes were mainly concentrated in TNF signaling pathway, MAPK signaling pathway, Apoptosis, FoxO signaling pathway, mTOR signaling pathway, and NOD-like receptor signaling pathway (Figure 1B). Further GO classification analysis of differentially expressed genes showed that the differentially expressed genes of A549 cells treated with α-MMC showed certain differences in various aspects, including biological processes, cell components, and molecular functions. There were significant differences in cell proliferation, cytoplasm, membrane, lysosome and so on (Figure 1C). The results of transcriptome differential analysis provide a reliable basis and a new idea for further research on the molecular mechanism of α-MMC exerting anti-tumor activity in this subject.

### 3.3 α-MMC significantly inhibited the proliferation of A549 and 95-D cells

**FIGURE 2:**
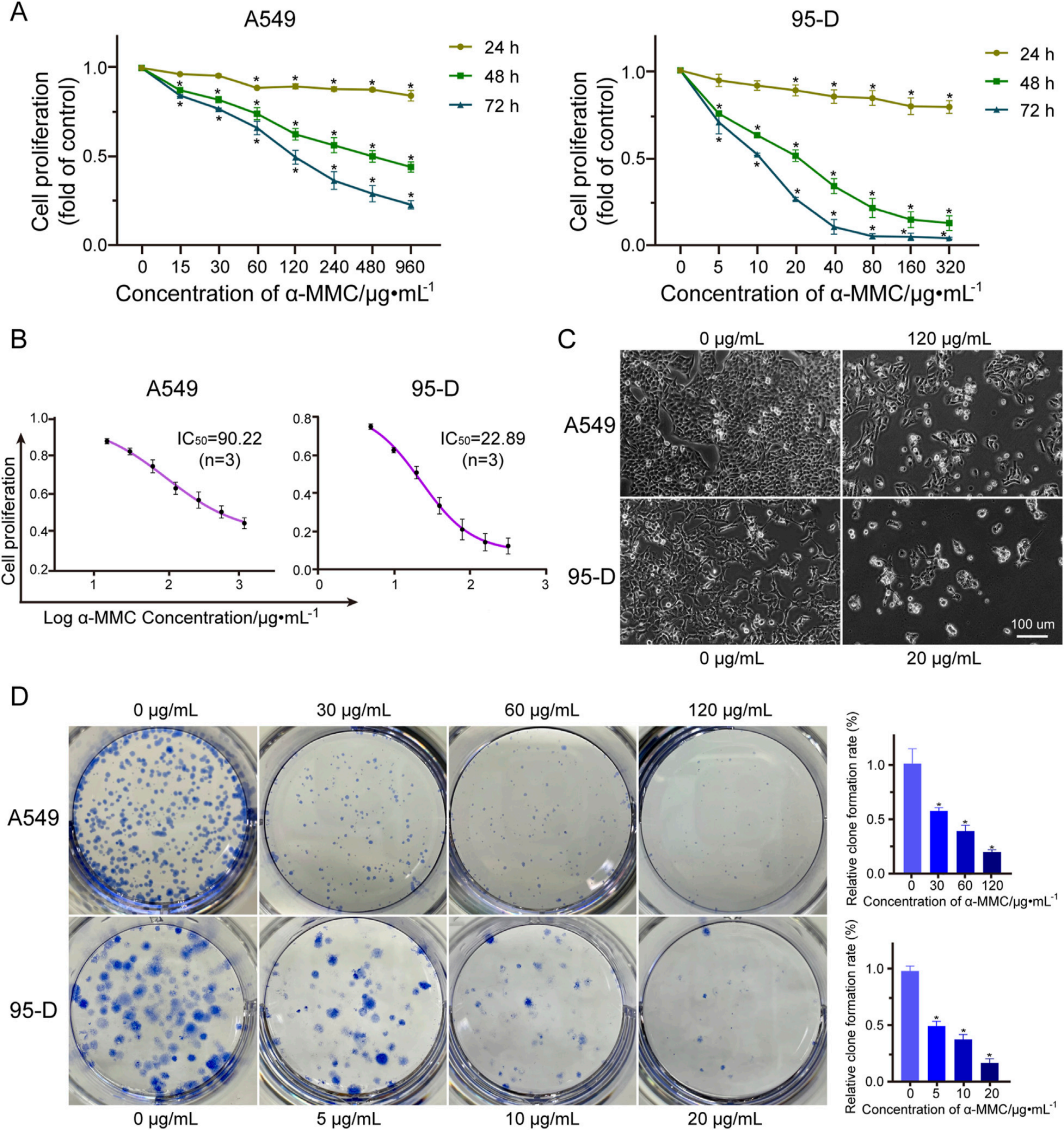
Effect of α-MMC on proliferation of A549 and 95-D cells **(A)**. The cell activity of A549 and 95-D cells treated with α-MMC for 24 h, 48 h and 72 h was measured by cck-8 kit; **(B)** The half-inhibitory concentration (IC50) was analyzed based on α-MMC intervention in A549 and 95-D cells for 48 h; **(C)** When α-MMC interfered with A549 and 95-D cells for 48 h, the cell morphology was observed by phase contrast microscopy (×100); **(D)** The colony formation ability of A549 and 95-D cells was observed after 14 days of treatment with different concentrations of α-MMC.

To investigate the effects of α-MMC on cell proliferation, we treated A549 and 95-D cells with different concentrations of α-MMC for 24, 48, and 72 h, respectively. The cell activity was measured by CCK-8 kit, and the results showed that α-MMC significantly inhibited the proliferation of A549 and 95-D cells in a time-dose-dependent manner (Figure 2A). Treatment with 480 μg/mL α-MMC for 72 h significantly inhibited the proliferation of A549 cells, and reduce the cell proliferation rate to (23.15 ± 1.89) %. Analysis of cell proliferation rate at 48 h showed that the semi-inhibitory concentration of α-MMC in A549 and 95-D cells was 90.22 μg/mL and 22.89 μg/mL, respectively (Figure 2B). Cell morphology was observed using phase-contrast microscopy (×100 magnification), we can see that after 48 h of α-MMC treatment, the cell growth is significantly inhibited, while cell atrophy, irregular shape, nucleopytosis and other apoptotic forms, and some even died (Figure 2C). In order to further confirm the effect of α-MMC on the proliferation of lung cancer cells, we explored the effect of α-MMC on cell clonal formation by clonal formation assay. As shown in Figure 2D, α-MMC induced a significant reduction in the number of A549 and 95-D cell clonal formation in a dose-dependent manner (P < 0.05). In summary, α-MMC showed excellent ability to inhibit the proliferation of lung cancer cells, significantly inhibiting cell proliferation and reducing cell viability in a time- and dose-dependent manner.

### 3.4 α-MMC effectively causes cell cycle arrest in A549 and 95-D cells in the G0/G1 or S phase

**FIGURE 3:**
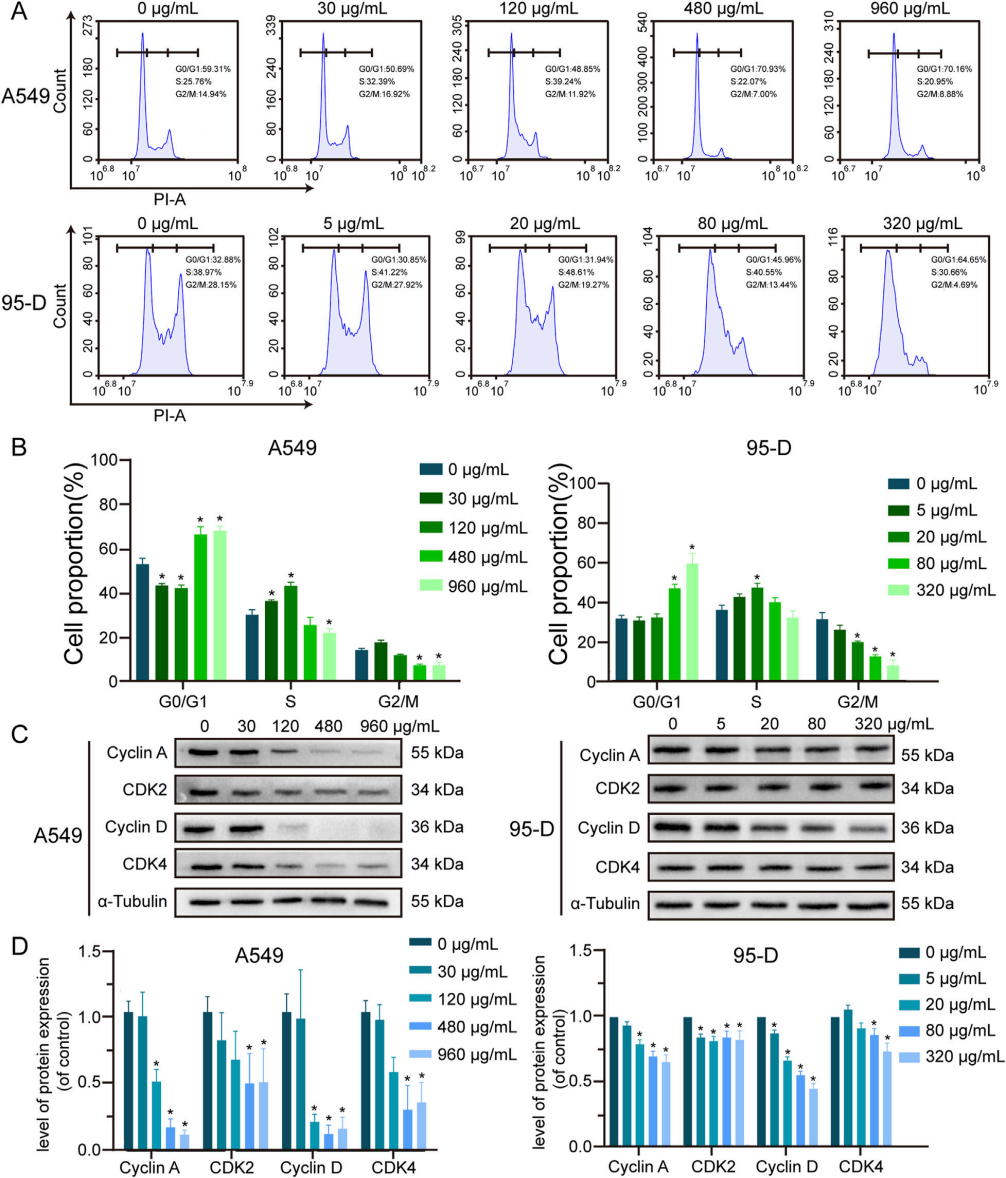
Effect of α-MMC on cell cycle in A549 and 95-D cells **(A)**. α-MMC interfered with A549 cells for 48 h and 95-D cells for 24 h respectively, and the cell cycle was analyzed by flow cytometry. **(B)** Calculate the percentage of cells in G0/G1, S and G2/M phases and represent them in the histogram. **(C)** Western blot detection of relevant cyclin levels. **(D)** Statistical analysis of Cyclin A, CDK2, Cyclin D and CDK4.

To investigate the effect of α-MMC on lung cancer cell cycle, PI staining was used to analyze the cell cycle by flow cytometry. As shown in Figures 3A,B, after treating A549 cells with different concentrations of α-MMC for 48 h, the proportion of S phase increased to (37.32 ± 0.45) % and (44.44 ± 1.33) % at low concentrations of α-MMC (30 and 120 μg/mL), respectively. Compared with the control group (30.95 ± 1.91) %, the proportion of cells in S phase was significantly increased (*P* < 0.05). However, the proportion of cells in the G0/G1 phase increased to (68.35 ± 2.97) % and (70.12 ± 1.69) % when treated with high concentrations of α-MMC (480 and 960 μg/mL), respectively. Compared with the control group (54.54 ± 2.19) %, the difference was statistically significant, showing G0/G1 phase cycle arrest (P < 0.05). At the same time, 95-D cells treated with different concentrations of α-MMC for 24 h showed the same trend of cycle arrest. S-phase cycle arrest was observed in cells treated with low concentration of α-MMC (5, 20 μg/mL), while G0/G1 phase cycle arrest was observed in cells treated with high concentration of α-MMC (80, 320 μg/mL).

In order to further determine the molecular mechanism of α-MMC causing cell cycle arrest in A549 and 95-D cells, we conducted an in-depth study of the Cyclin-CDK-CKI signaling pathway using western blotting. The results showed that the expression levels of S-phase cycle regulatory proteins (Cyclin A, CDK2) and G0/G1 phase cycle regulatory proteins (Cyclin D, CDK4) were downregulated (Figures 3C,D), and the protein expression decreased more significantly in the high-concentration α-MMC intervention group. Among them, Cyclin D-CDK 4/6 and Cyclin E-CDK 2 complexes sequentially initiate G1/S phase transformation, and Cyclin A-CDK 2 complexes guarantee DNA replication in S phase. Therefore, the decreased expression levels of Cyclin D-CDK 4 and Cyclin A-CDK 2 were confirmed by the previous flow detection results. It is suggested that α-MMC can downregulate the key protein mediating the G1 and S phase transition through the Cyclin-CDK regulatory pathway, and thus effectively induce the cycle arrest of lung cancer cells. The rule is reflected in that when the concentration is low, the cell cycle is blocked in S phase, and with the increase of the intervention concentration, the cells present G0/G1 phase cycle arrest.

### 3.5 α-MMC inhibits cell migration and invasion of A549 and 95-D cells

**FIGURE 4:**
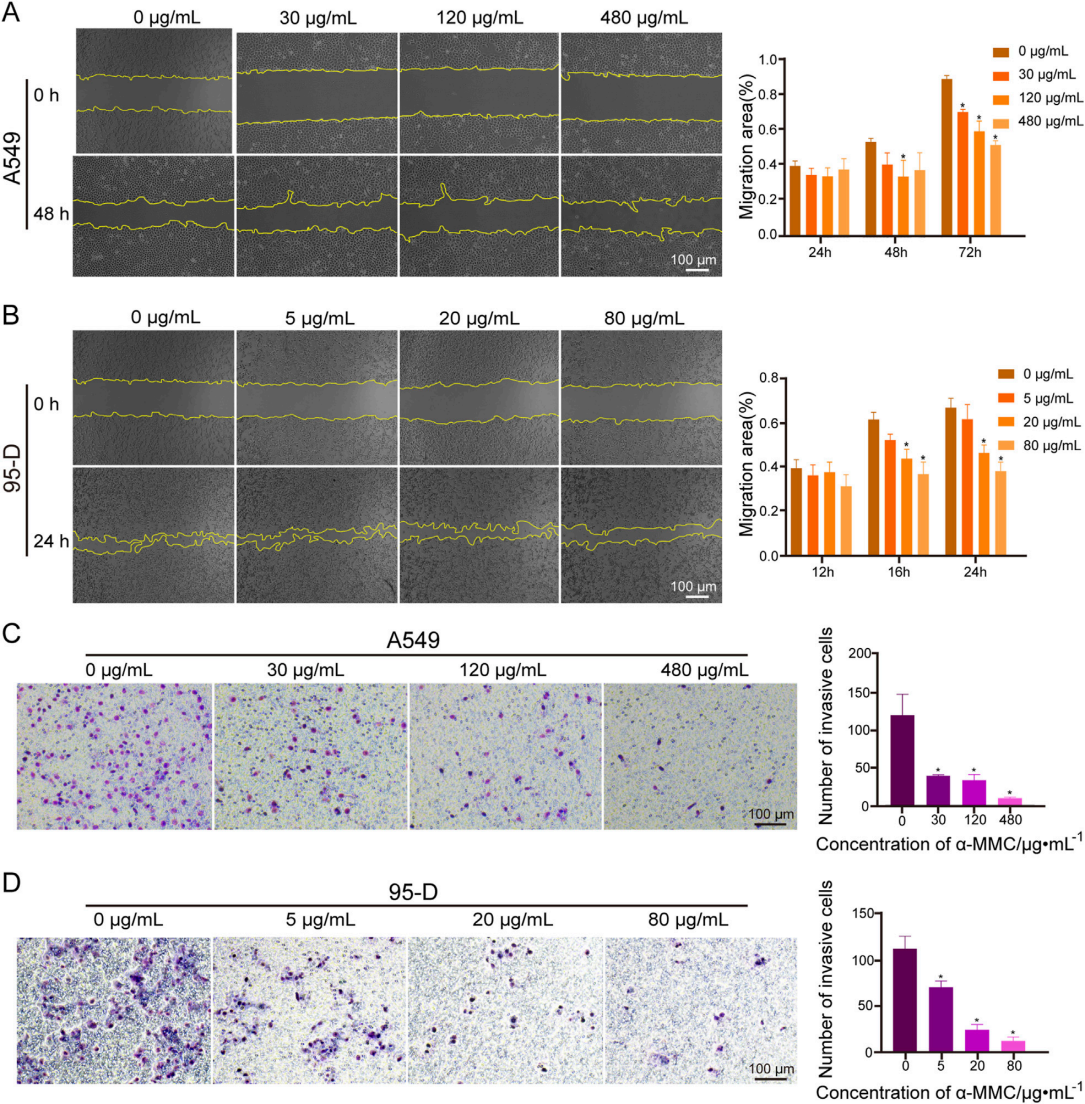
Effects of α-MMC on cell migration and invasion in A549 and 95-D cells **(A,B)**. α-MMC interfered with A549/95-D cells, and the cell migration was observed and recorded under the microscope (×40) at the same location at different time points, and displayed in the histogram; **(C,D)** A549 and 95-D cells were treated with different concentrations of α-MMC for 48 h, and the influence of α-MMC intervention on cell invasion was evaluated by Transwell method. The number of cells passing through the chamber was observed in 5 fields randomly selected under the microscope (×100), and displayed in the histogram.

The effect of α-MMC on the migration of lung cancer cells was investigated by scratch test. A549 and 95-D cells were treated with different concentrations of α-MMC, and the migration area of cells was observed at different time points. As shown in Figure 4A, when α-MMC treated A549 cells for 72 h, cell mobility after α-MMC treatment (30, 120, and 480 μg/mL) was (71.54 ± 1.26) %, (60.53 ± 4.74) %, and (52.81 ± 1.97) %, respectively. Compared with the control group (90.31 ± 1.59) %, the cell migration area was significantly decreased (*P* < 0.05). At the same time, α-MMC inhibited the migration of 95-D cells more significantly in the group treated with 95-D cells, and the cell mobility decreased significantly after only 24 h of intervention. The cell mobility of the control group and the 5, 20, and 80 μg/mL α-MMC intervention groups were (67.39 ± 3.50) %, (62.25 ± 5.36) %, (46.86 ± 2.94) %, (38.54 ± 3.32) %, respectively (Figure 4B). In conclusion, α-MMC can effectively inhibit the migration of lung cancer A549 and 95-D cells in a dose-dependent manner.

In the transwell experiment, we simulated cell membranes with Matrigengel to explore the effect of α-MMC on lung cancer cell invasion. A549 and 95-D cells were treated with different concentrations of α-MMC for 48 h, and the transmembrane cells were counted. In the A549 cell treatment group, there were (120 ± 23), (39 ± 1), (34 ± 6) and (10 ± 1) penetrating cells in the control group and α-MMC intervention group (30, 120, and 480 μg/mL), respectively (Figure 4C). In the 95-D cell treatment group, there were (112 ± 11), (70 ± 6), (25 ± 5), and (13 ± 3) penetrating cells in the control group and the 5, 20, and 80 μg/mL α-MMC intervention groups, respectively (Figure 4D). Compared with the control group, α-MMC can significantly inhibit the invasion of lung cancer A549 and 95-D cells in a dose-dependent manner.

### 3.6 α-MMC significantly induced apoptosis of A549 and 95-D cells

**FIGURE 5:**
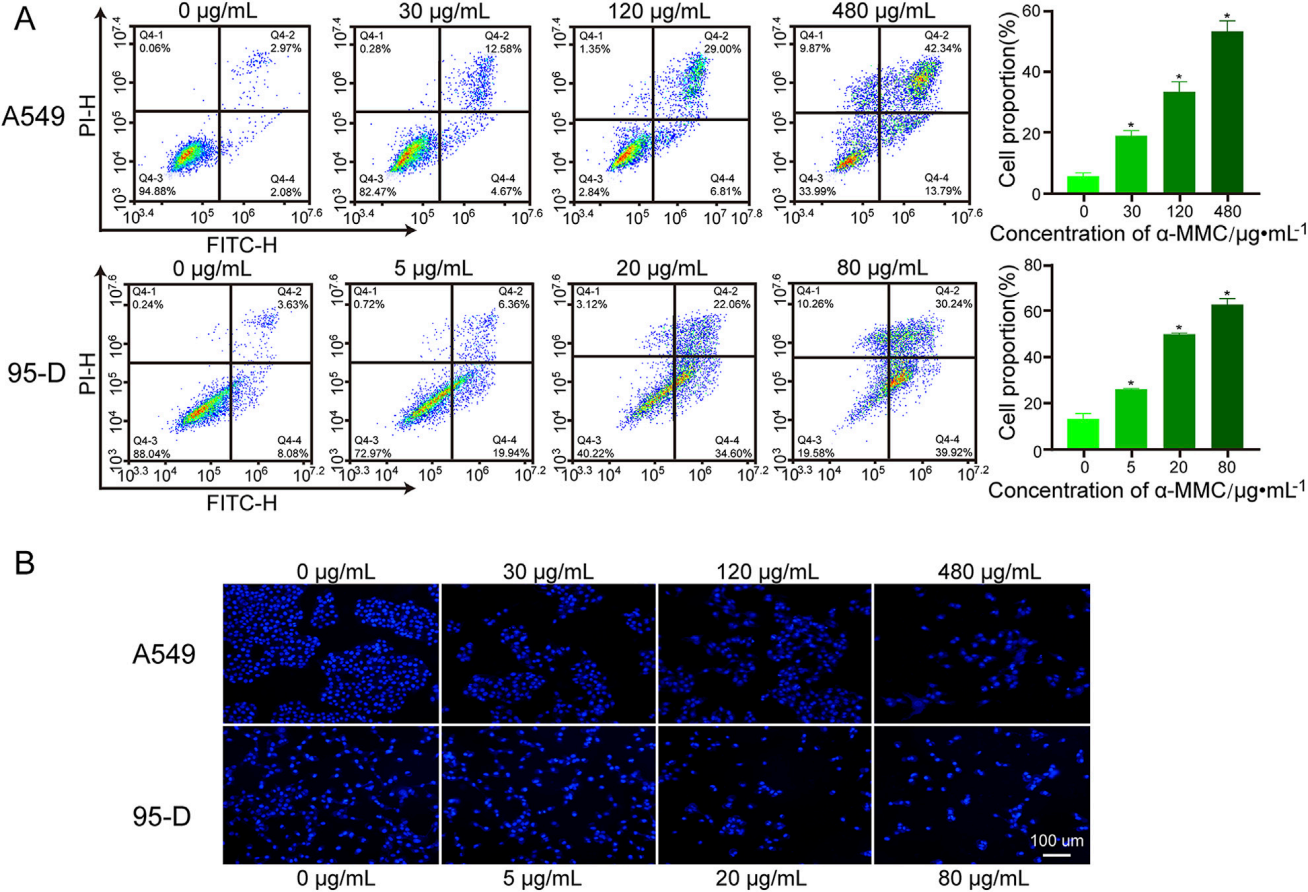
Effect of α-MMC on apoptosis of A549 and 95-D cells. α-MMC interfered with A549 cells for 72 h and 95-D cells for 48 h, respectively. **(A)** The percentage of apoptosis was detected by PI/Annexin V-FITC double staining and expressed in the histogram. **(B)** Hoechst 33,342 staining was used to observe apoptotic cells.

To determine whether α-MMC can induce tumor cell apoptosis, we employed Annexin V-FITC/PI double staining in conjunction with flow cytometry to detect apoptosis in A549 and 95-D cells. In Figure 5A, apoptotic density distribution shows dead or late apoptotic cells in the upper right (UR) region, early apoptotic cells in the lower right (LR) region, and normal cells in the lower left (LL) region. Therefore, the total apoptosis rate was the proportion of UR + LR. The results showed that the total apoptosis rate of A549 cells was significantly increased in a dose-dependent manner after 72 h treatment with α-MMC. Compared with (5.89 ± 0.81) % in the control group, the proportions of total apoptotic cells in the 30, 120 and 480 μg/mL α-MMC treatment groups were (19.04 ± 1.39) %, (33.36 ± 2.72) %, and (53.16 ± 2.82) %, respectively. Similarly, after α-MMC treatment for 48h, the apoptosis rate of 95-D cells increased significantly, reaching (25.97 ± 0.30) %, (49.77 ± 0.43) % and (62.65 ± 2.11) %, respectively, which was significantly different from (13.18 ± 1.88) % in the control group.

Nuclear shrinkage and chromatin aggregation are typical features of apoptosis, so we further observed the changes of nuclei by Hoechst staining. With the help of fluorescence microscopy, we observed that A549 and 95-D cells treated with α-MMC showed enhanced fluorescence signal, nuclear contraction, nuclear membrane depression, and nuclear fragmentation (Figure 5B). Therefore, α-MMC significantly induced apoptosis of A549 and 95-D cells in a dose-dependent manner by Annexin V-FITC/PI double staining and Hoechst staining.

### 3.7 α-MMC induced increased ROS levels and decreased MMP levels in A549 and 95-D cells

**FIGURE 6:**
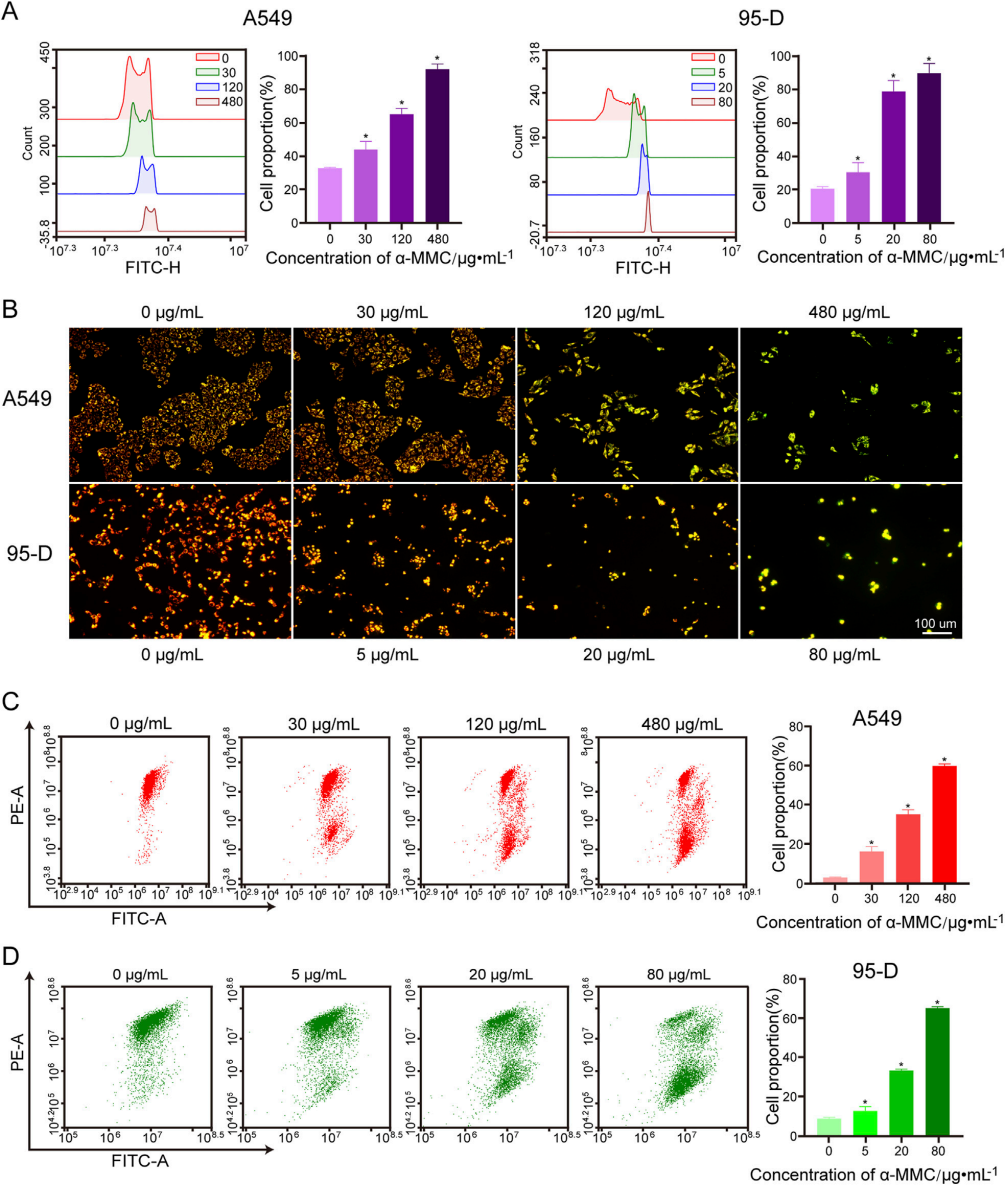
Effect of α-MMC on mitochondrial membrane potential in A549 and 95-D cells. α-MMC interfered with A549 cells for 72 h and 95-D cells for 48 h, respectively. **(A)** Cell ROS levels were measured by flow cytometry. **(B)** The changes of MMP after JC-1 staining were observed by fluorescence microscopy. **(C,D)** MMP was analyzed by flow cytometry and displayed in the histogram.

It has been reported that Reactive oxygen species (ROS) play an important role in cell apoptosis and cell cycle arrest. As shown in Figure 6A, intracellular ROS levels were positively correlated with α-MMC intervention concentration in cells. After 72 h of treatment with α-MMC at different concentrations (30, 120, 480 μg/mL), ROS levels in A549 cells reached (43.94 ± 4.04) %, (65.17 ± 2.74) %, and (91.98 ± 2.58) %, respectively, which were significantly higher than that in the control group (32.84 ± 0.46) % (*P* < 0.05). Similarly, the same trend was observed in the 95-D cell treatment group, and ROS levels increased significantly with the increase of α-MMC intervention concentration. These results indicated that α-MMC induced apoptosis of A549 and 95-D cells may be related to the overproduction of ROS.

The substrate end of the respiratory chain in the inner mitochondrial membrane is one of the main sources of ROS production. To investigate the source of ROS, we further studied mitochondrial membrane potential by flow cytometry and fluorescence microscopy using JC-1 fluorescent probes. When the mitochondrial membrane potential is high, JC-1 aggregates in the mitochondrial matrix as polymers, emitting red fluorescence. Conversely, when the membrane potential decreases, JC-1 exists in the cytoplasm as monomers, emitting green fluorescence. We observed that A549 and 95-D cells in the untreated group formed JC-1 polymers and fluoresced in red, while A549 and 95-D cells treated with different concentrations of α-MMC fluoresced in green under JC-1 treatment (Figure 6B). This result indicates that α-MMC can cause a decrease in mitochondrial membrane potential. In flow cytometry, we also observed that the mitochondrial membrane potential (MMP) of A549 and 95-D cells treated with different concentrations of α-MMC decreased in a dose-dependent manner. (Figures 6C,D). Therefore, the treatment of alpha-MMC may lead to the accumulation of ROS in mitochondria, and the elevated ROS levels may be related to mitochondrial signaling pathways.

In summary, we found that α-MMC can cause oxidative stress in lung cancer A549 and 95-D cells, resulting in a sharp increase in ROS levels. Combined with significant depolarization of mitochondrial membrane potential, we speculated that the increased ROS is highly likely to come from the mitochondrial respiratory chain.

### 3.8 α-MMC induced apoptosis of A549 cells through mitochondrial pathway

**FIGURE 7:**
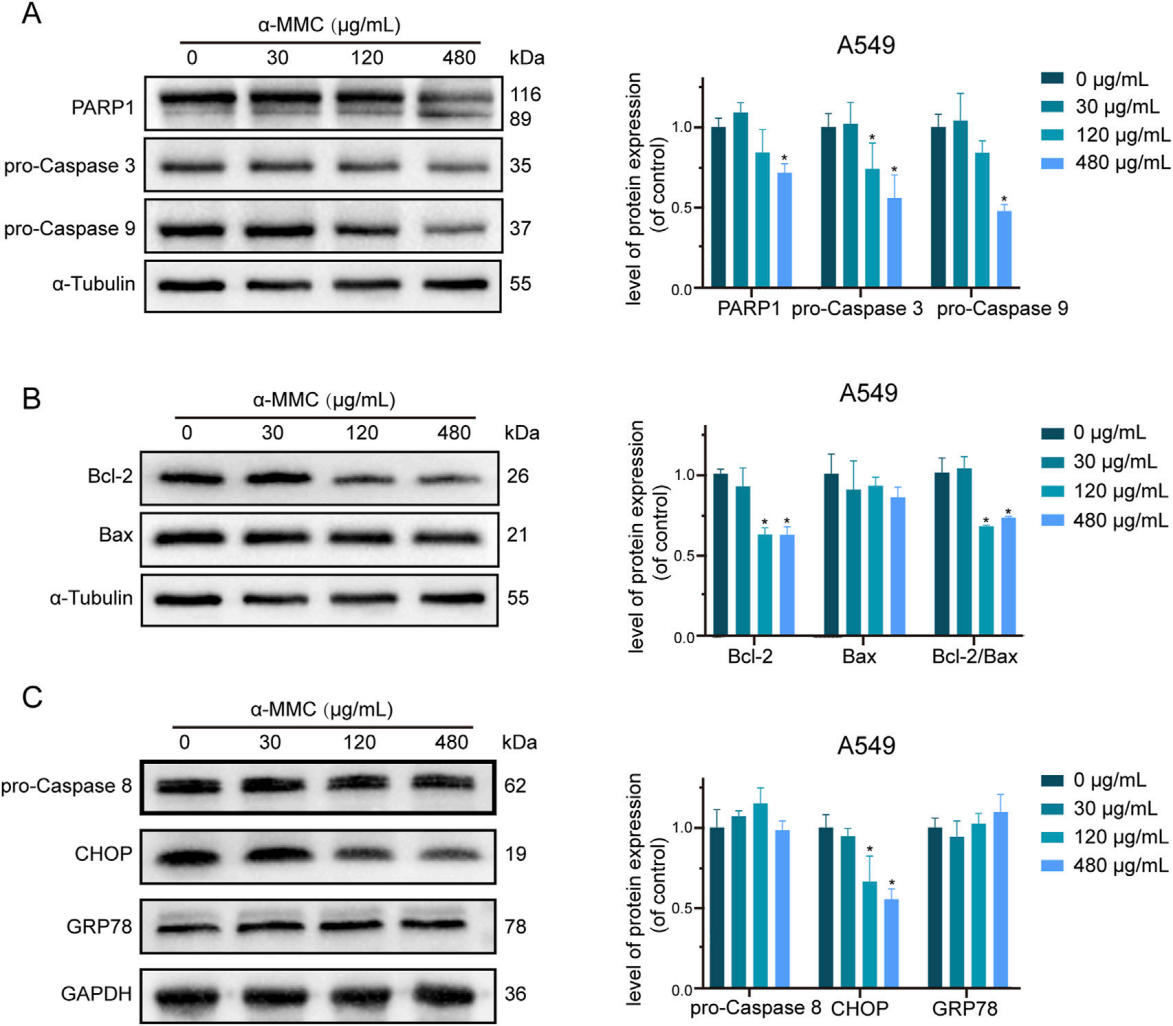
Study on apoptosis pathway of A549 cells induced by α-MMC. α-MMC interfered with A549 cells for 72 h, and the expression of apoptotic pathway protein was detected by WB. **(A)** apoptotic classical proteins; **(B)** Mitochondrial pathway proteins; **(C)** Endoplasmic reticulin pathway proteins. Note: A control image α-Tubulin is reused in Figures 7A,B for illustration purposes.

In order to further explore the regulatory mechanism of α-MMC induced apoptosis in lung cancer cells, we conducted an in-depth study on the classical proteins involved in apoptosis. The results showed that with the increase of α-MMC intervention concentration in A549 cells, the expression levels of pro-Caspase 3 and pro-Caspase 9 gradually decreased (Figure 7A). At the same time, due to the presence of Caspase-3 cutting sites on PARP1, Caspase-3 activation can cut PARP1 into fragments of different sizes. In Figure 7A, it was observed that the expression level of PARP1 in 116 kDa was gradually decreased, while that of PARP1 in 89 kDa after being clipped was gradually increased, which further indicated that Caspase 3 was successfully activated in the cell and effectively played its biological activity to induce cell apoptosis.

The Caspase cascade is activated mainly through three pathways, namely, mitochondrial pathway, endoplasmic reticulum pathway and death receptor ligand pathway. As shown in Figure 7B, the expression level of Bcl-2, a key protein regulating the mitochondrial pathway, gradually decreased, while the ratio of Bcl-2/Bax significantly decreased. Since the ratio of Bcl-2/Bax plays an important role in determining the sensitivity of apoptosis, the mitochondrial pathway is involved in the regulation of apoptosis. However, no significant changes were detected in GRP78 and Caspase 8, key proteins that regulate the endoplasmic reticulum pathway and the death receptor ligand pathway, respectively (Figure 7C). Therefore, whether the endoplasmic reticulum pathway and the death receptor ligand pathway can induce apoptosis needs to be further studied.

In conclusion, studies have shown that α-MMC mainly inhibits the expression of Bcl-2 through the mitochondrial pathway to activate the Caspase cascade, and significantly induces apoptosis of lung cancer cells through activation of effector protein caspase-3 and induction protein caspase-9.

### 3.9 α-MMC induces apoptosis of A549 cells through TNF signaling pathway

**FIGURE 8 F8:**
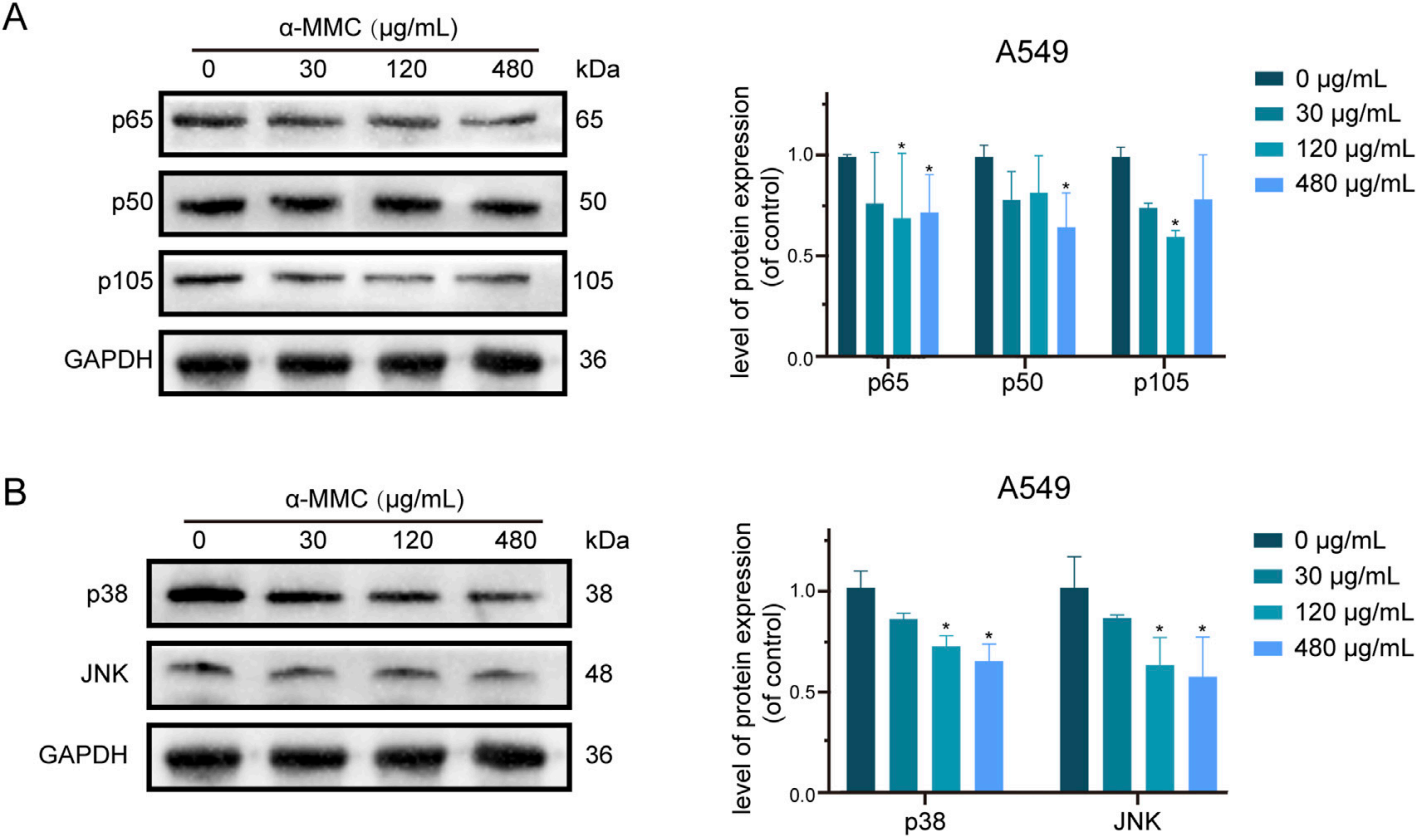
Study on TNF signaling pathway in A549 cells induced by α-MMC. α-MMC interfered with A549 cells for 72 h, and the expression of TNF pathway protein was detected by WB. **(A)** NF-κB signaling pathway; **(B)** MAPK signaling pathway. Note: A control image GAPDH is reused in Figures 7C,8A,B, [Fig F8] for illustration purposes.

Based on the analysis of KEGG enrichment of differentially expressed genes in transcriptome sequencing, it was suggested that the most significant enrichment was TNF signaling pathway, so we conducted an in-depth study on the TNF signaling pathway. The TNFR1 receptor is mainly controlled by two pathways, NF-κB and MAPK. The NF-κB pathway is involved in cell apoptosis and survival by inducing p65 phosphorylation and binding with p50 to form a two-source complex. As shown in Figure 8A, we found that the expression levels of p65, p50 and p105, a precursor protein of p50, were reduced in A549 cells under the intervention of α-MMC. These results indicated that both p65 and p50 were activated in the cell, which mediated the regulation of TNF signaling pathway through NF-κB pathway.

As another pathway to activate TNFR1 receptor, MAPK signaling pathway plays an important role in cell proliferation, differentiation, inflammatory response and apoptosis (Zhang and Liu, 2002). As a key molecule of MAPK pathway, p38/JNK is closely related to apoptosis, survival signal transduction, and cell stress response (Morrison, 2012; Canovas and Nebreda, 2021). As shown in Figure 8B, the expression levels of p38/JNK in A549 cells after α-MMC intervention were significantly reduced, and the protein expression decreased more significantly with the increase of intervention concentration. Therefore, MAPK pathway can mediate TNF signaling pathway to participate in the regulation of cell proliferation, cycle and apoptosis. In conclusion, these results suggest that α-MMC mediates the TNF signaling pathway through both NF-κB (downregulating p65/p50) and MAPK (downregulating p38/JNK) signaling pathways to regulate cell processes, and then activate the caspase cascade to induce apoptosis of A549 cells.

## 4 Discussion

In recent years, more and more attention has been paid to the prevention and treatment of tumor by traditional Chinese medicine (Yin et al., 2008). Bitter gourd, as a common traditional Chinese medicine, has various biological activities such as lowering blood sugar, lowering blood lipids, antioxidant, anti-inflammatory and anticancer (Raina et al., 2016; Jia et al., 2017). Studies have reported that α-MMC and MAP30, two type I ribosome-inactivating proteins (RIPs) derived from bitter melon, effectively inhibit the growth of various tumor cell lines and exhibit broad-spectrum antitumor activity (Chan et al., 2020; Zhang et al., 2020). These proteins demonstrate promising application prospects in antitumor research due to their potent and versatile anticancer properties (Manoharan et al., 2014). Our laboratory took α-MMC as raw material and lung cancer cell lines A549 and 95-D as research objects to conduct a comprehensive exploration on its anti-tumor activity and mechanism of action, aiming to develop the plant-derived type I ribosome-inactivating protein α-MMC as a new clinical anti-tumor drug.

In our study of A549 and 95-D cells by α-MMC, we found that α-MMC can significantly inhibit the proliferation of lung cancer cells in a time- and dose-dependent manner in various aspects, including cell proliferation, morphology, and clonal formation. In the study of the semi-inhibitory concentration of cell activity, it was found that very low concentration of α-MMC can cause significant changes in 95-D cells. It can be seen that α-MMC has different sensitivity to different cell lines, especially to 95-D cells, about 4 times that of A549 cells. In the study of cell cycle, G0/G1 phase and S phase are the key periods for protein synthesis and DNA replication, and a large amount of RNA and protein need to be synthesized to provide sufficient raw materials for DNA replication (Maddika et al., 2007). Given its *N*-glycosidase activity, α-MMC likely disrupts protein synthesis within cells. Therefore, α-MMC can effectively block the cell cycle in the G0/G1 phase or the S phase, and this result matches with its biochemistry. Similarly, cycle arrest must inhibit normal cell proliferation, which further explains the phenomenon that α-MMC inhibits cell proliferation.

One of the key factors in the death of cancer patients is the migration and invasion of tumor cells, and understanding the mechanism of cell migration is very important for cancer research (van de Merbel et al., 2018). As a highly metastatic cell line of lung cancer, 95-D cells had a very significant effect of α-MMC on inhibiting their migration in scratch experiments, and the trend of inhibiting migration was significantly observed after only 24 h of treatment. However, although α-MMC interfered with A549 cells for 48 h, the inhibitory effect was not obvious, which may be due to the weak migration ability of A549 cells, so they failed to reflect the intervention effect of α-MMC. Tumor cell invasion is an important parameter in oncology research, which can reflect the influence of tumor-related signaling pathways, drug therapy, targeted therapy, and oncogenic or tumor suppressor genes (Mayor and Etienne-Manneville, 2016; Aras et al., 2018). Therefore, focusing on its mechanism in future studies will have more important significance for various physiological and pathological processes.

More and more evidence showed that tumor cell death induced by chemotherapy drugs is always related to apoptosis (Xu et al., 2019). In this study, we found that α-MMC induced apoptosis of A549 and 95-D cells with typical cell morphological changes. Combined with CCK-8 results, it was found that the proportion of apoptotic cells was consistent with the proliferation of lung cancer cells after α-MMC intervention. These results demonstrated that the inhibitory effect of α-MMC on the proliferation of A549 and 95-D cells was closely related to the occurrence of apoptosis. In this experiment, we detected significantly increased ROS levels and MMP depolarization in A549 and 95-D cells, and mitochondria are one of the main sources of ROS production, suggesting that α-MMC-induced apoptosis may be related to the mitochondrial pathway (Luo et al., 2019).

In order to confirm the previous conjecture, we conducted an in-depth study on the relevant signaling pathways that induce apoptosis. It was found that α-MMC mainly activated the Caspase cascade through the mitochondrial pathway, and effectively induced apoptosis through the classical effector protein Caspase-3 and the induction protein Caspase-9. Bcl-2 can be inserted into the mitochondrial membrane to form ion channels and change the permeability of the membrane (Willis et al., 2003). Therefore, the decreased expression level of Bcl-2 in lung cancer cells after α-MMC intervention exactly explains the phenomenon of depolarization of mitochondrial membrane potential of lung cancer cells, which is consistent with previous results. However, intracellular proapoptotic activity mainly depends on cleaved-caspase-3 and cleaved-caspase-9 after activation (Fan et al., 2005). This study only showed the expression level of zymogen in cells, and the results were one-sided. Therefore, it will be more convincing if the expression of cleaved-caspase3 and cleaved-caspase9 can be further detected in subsequent studies.

The TNF signaling pathway is an important cytokine regulating apoptosis, survival, inflammation and other mechanisms, which is mainly regulated by two receptors, TNFR1 and TNFR2 (Pasquereau et al., 2017). TNFR1 receptor is controlled by NF-κB and MAPK pathways, and is involved in apoptosis and necrosis. This study confirmed that the TNF signaling pathway plays a crucial role in the apoptosis of lung cancer A549 cells through the co-regulation of NF-κB and MAPK pathways. The KEGG classification results of translibrary sequencing of α-MMC in the previous stage suggested that TNF signaling pathway was the most significant pathway in the differential genes, which was consistent with the results in transcriptome sequencing. The downregulated expression of p65, p50, p38 and JNK confirmed that NF-κB and MAPK pathways were involved in the regulation of cell proliferation and apoptosis (Webster and Vucic, 2020). However, the biological activity of the cell is mainly through phosphorylated proteins, so it will be more clinical significance to further explore the phosphorylated target proteins in subsequent studies.

In recent years, a number of studies have been conducted on the anti-tumor activity of α-MMC, and it has been found that it has broad-spectrum anti-tumor activity against A549, Hela, HepG-2 and other tumor cells, and is expected to be used as a new and relatively safe drug for the prevention and treatment of lung cancer (Farooqi et al., 2018). However, as exogenous proteins, RIPs exhibit strong antigenicity in humans, large toxic side effects, short plasma half-life, and may cause adverse reactions such as anaphylactic shock during clinical application, which limits their clinical application (Stirpe et al., 1992). Polyethylene glycol (PEG) has the excellent characteristics of low toxicity, low immunogenicity and water solubility (Meng et al., 2012). Therefore, modifying RIPs with small molecule PEG can increase drug stability, prolong metabolic half-life *in vivo*, and reduce protein immunogenicity and toxic side effects (Wang et al., 2004). It is one of the possible ways to solve this problem, but the specific mechanism remains to be further explored. Moreover, natural α-MMC lacks a cell-targeting domain and relies on exogenous carriers (such as antibodies or ligands) to achieve tumor targeting. Effectively addressing the above issues will enable better clinical application of α-MMC. In conclusion, we demonstrate here that α-MMC has excellent antitumor activity and may be a promising new drug for the treatment of non-small cell lung cancer. However, this preliminary study has certain limitations, such as the safety of the active ingredients of α-MMC and the use of animal models *in vivo*, which need to be further studied.

## 5 Conclusion

Our research group successfully extracted α-MMC 56.61 mg from bitter melon seeds with a yield of 0.92%, and carried out a series of studies on the anti-tumor activity and mechanism of α-MMC. The results showed that α-MMC showed excellent antitumor activity against A549 and 95-D cells. α-MMC can significantly inhibit the proliferation of A549 and 95-D cells, block the cell cycle in G0/G1 phase or S phase, significantly induce lung cancer cell apoptosis, inhibit cell migration and invasion, and induce intracellular ROS level increase and MMP depolarization in a dose-dependent manner. Mechanism studies further demonstrated that α-MMC downregulates the expression of Cyclin D, CDK4, Cyclin A and CDK2 through cyclin-CDK-CKI signaling pathway, thereby participating in the regulation of cell cycle. α-MMC activates the Caspase cascade through mitochondrial pathway, and co-mediates the TNF signaling pathway through NF-κB and MAPK signaling pathways, thereby activating downstream effector factors of Caspase and inducing apoptosis of tumor cells. In conclusion, α-MMC shows excellent antitumor activity and may be developed as a new clinical antitumor drug.

## Data Availability

The original contributions presented in the study are publicly available. This data can be found from the NCBI database, accession number PRJNA1272486.
