# Supplementary material for: The type I ribosome-inactivating protein α-MMC induced significant apoptosis of lung cancer A549 and 95-D cells by activating the caspase cascade through TNF signaling pathway
Source: Front Pharmacol. 2025 Jun 9;16:1529151. doi: 10.3389/fphar.2025.1529151 (PMC12183754; doi:10.3389/fphar.2025.1529151)

Supplementary Material

1 Graphical Abstract

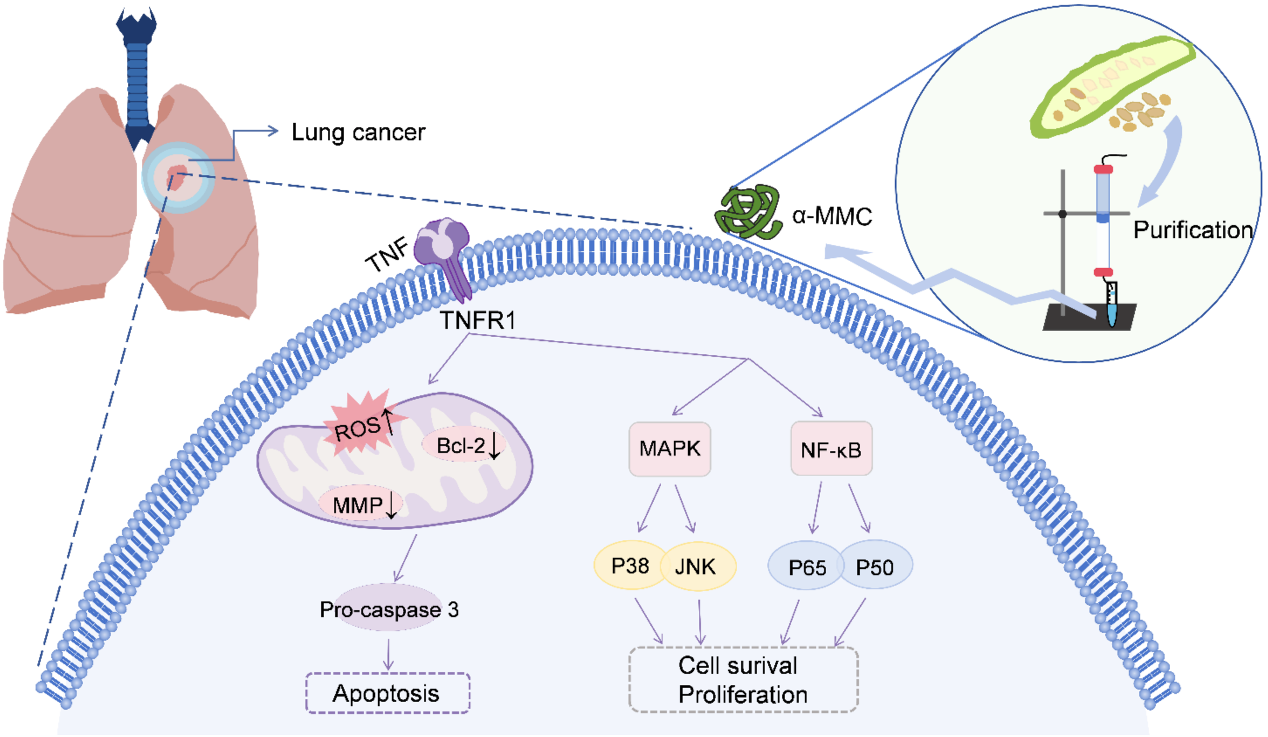

2 Supplementary Figures and Tables

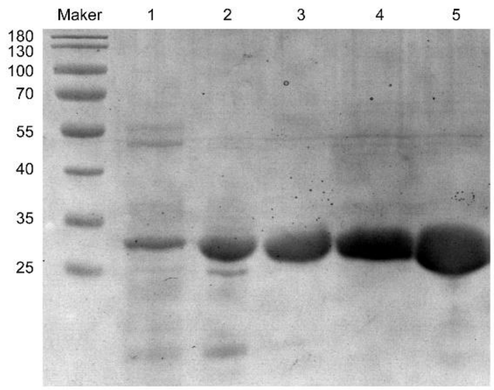

Figure S1. The profile of different purification stages by SDS-PAGE. Lane 1: Crude extracts; Lane 2: 30~60% A.S. precipitation; Lane 3: SP-Sepharose FF chromatography; Lane 4: Sephacryl S-100 chromatography; Lane 5: SP-Sepharose FF chromatography

Table S1. Purification of α-MMC from bitter melon seeds.

Steps	Total volume (mL)	Protein conc. (mg/mL)	Protein content (mg)	Recovery of Proteins (%)
Crude extracts	375	-	-	-
pH3.6 treatment	375	16.39	6146.25	100
30~60% A.S. precipitation	90	14.39	1295.10	21.07
SP-Sepharose FF chromatography	110	4.02	442.20	7.19
Sephacryl S-100 chromatography	208	0.96	199.68	3.25
SP-Sepharose FF chromatography	9	6.29	56.61	0.92

Note: Values reported are an average value of three preparations. The data of Protein content was quantified using the BCA kit.

3 Raw data from Western Blots

3.1 Uncropped Western Blots of A549 cells about cell cycle regulatory proteins

Cyclin A

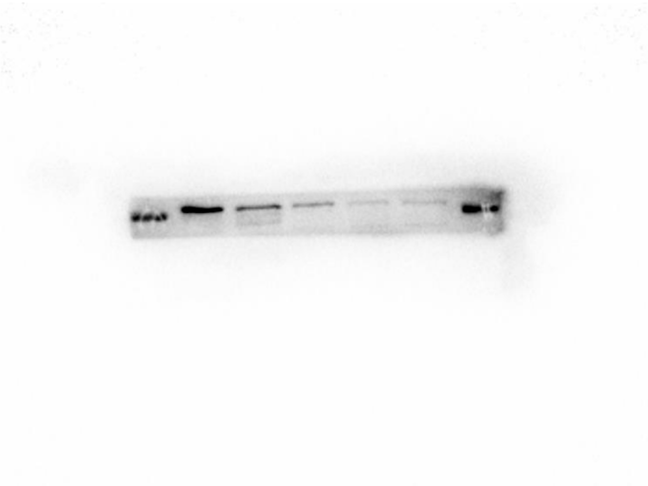

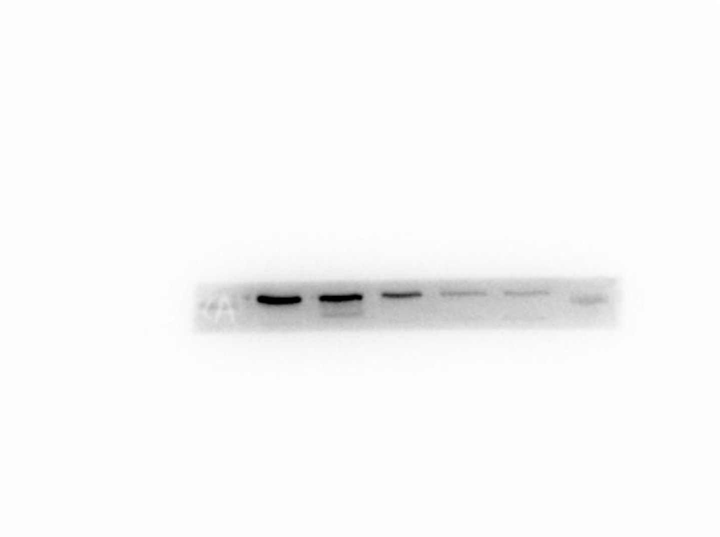

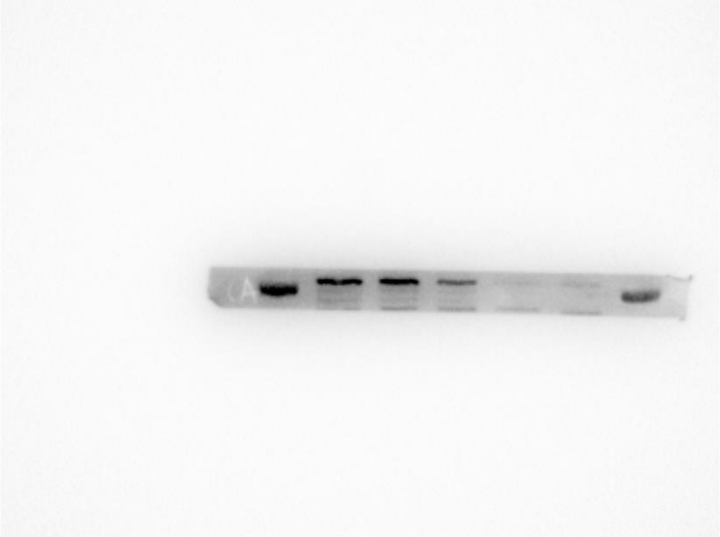

CDK2

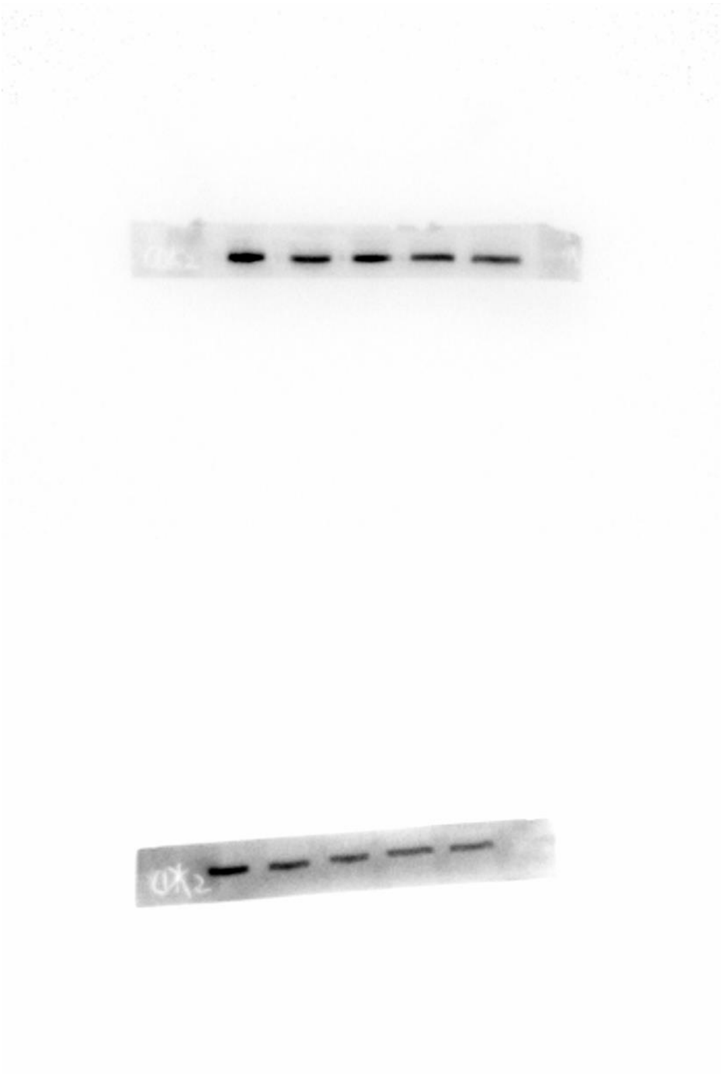

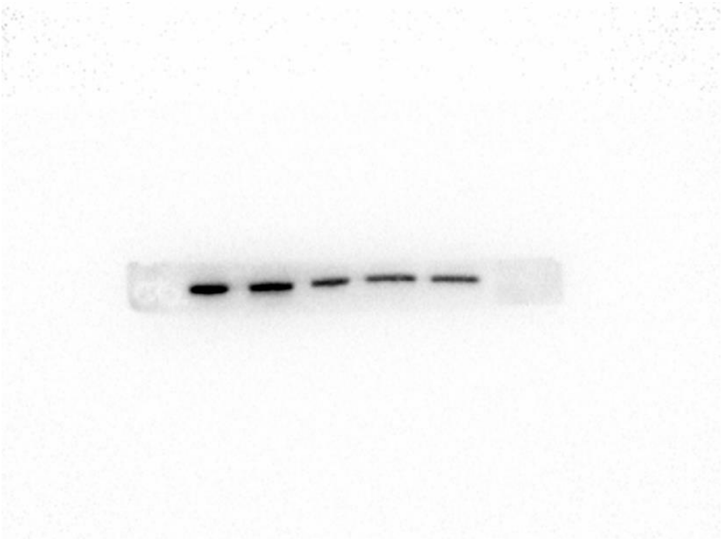

Cyclin D

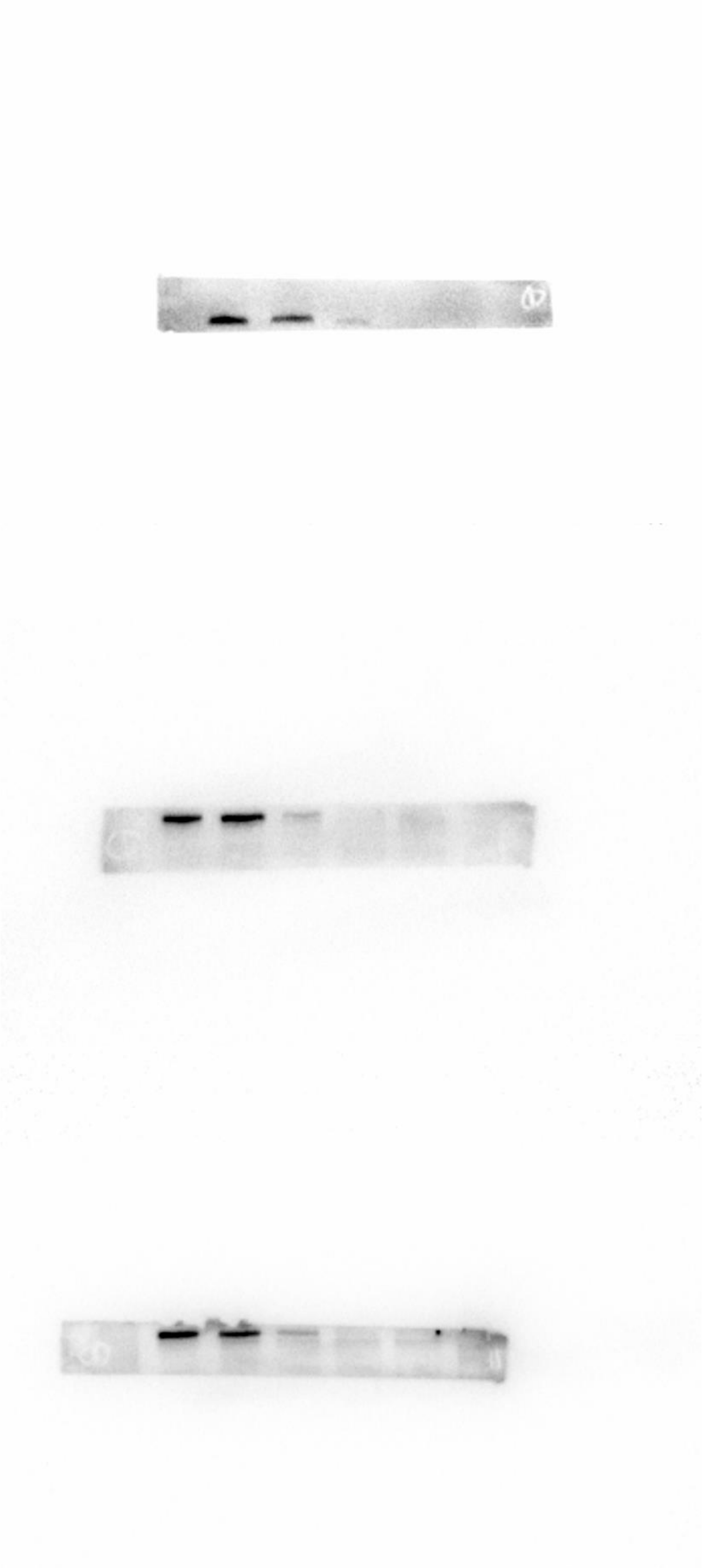

CDK4

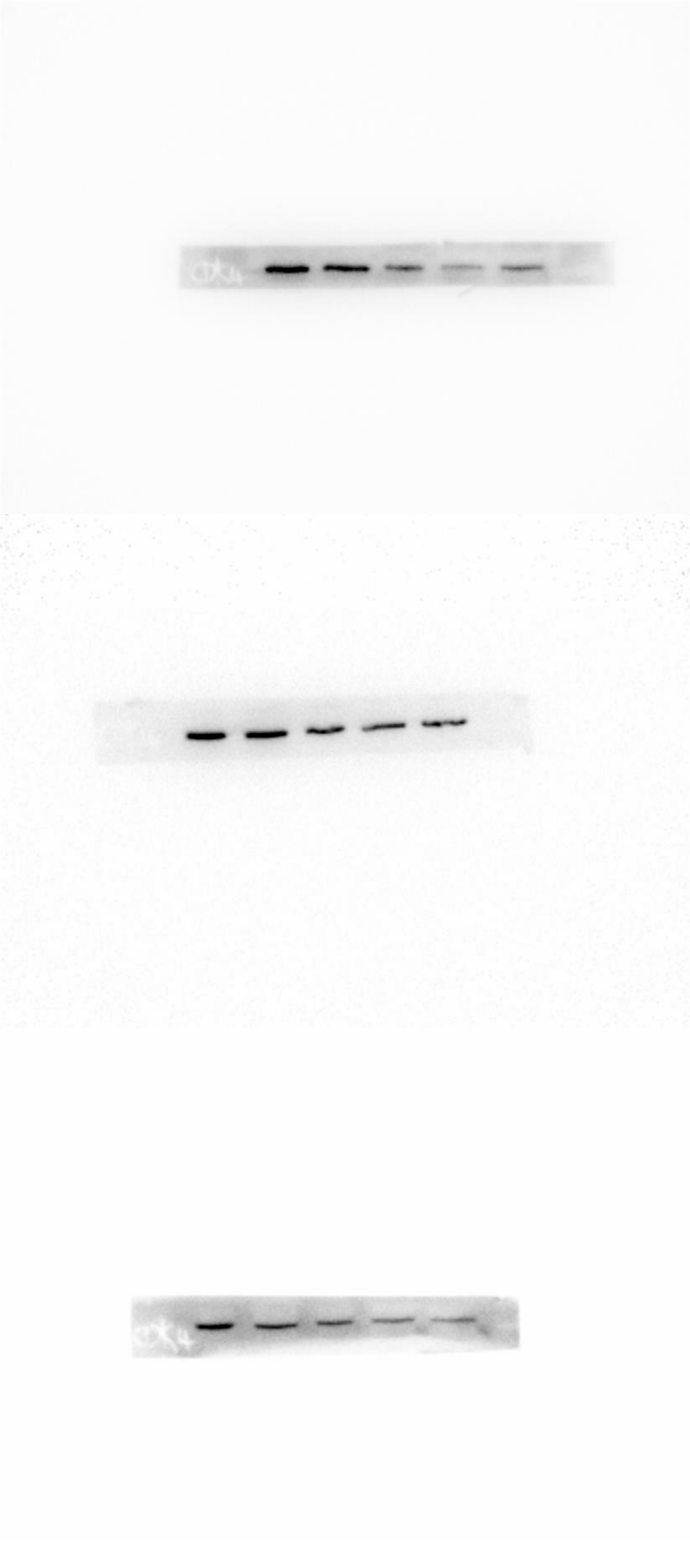

α-Tubulin

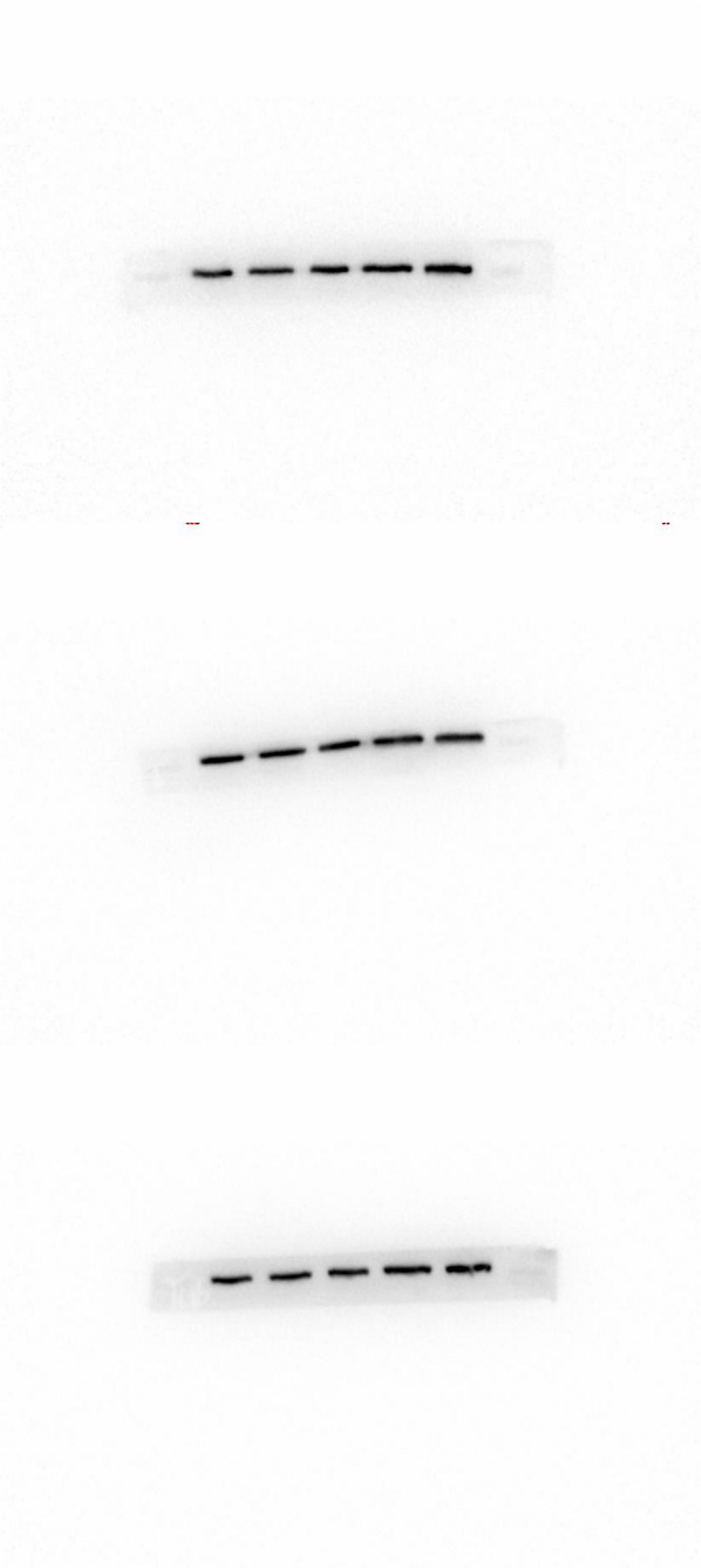

3.2 Uncropped Western Blots of 95-D cells about cell cycle regulatory proteins

Cyclin A

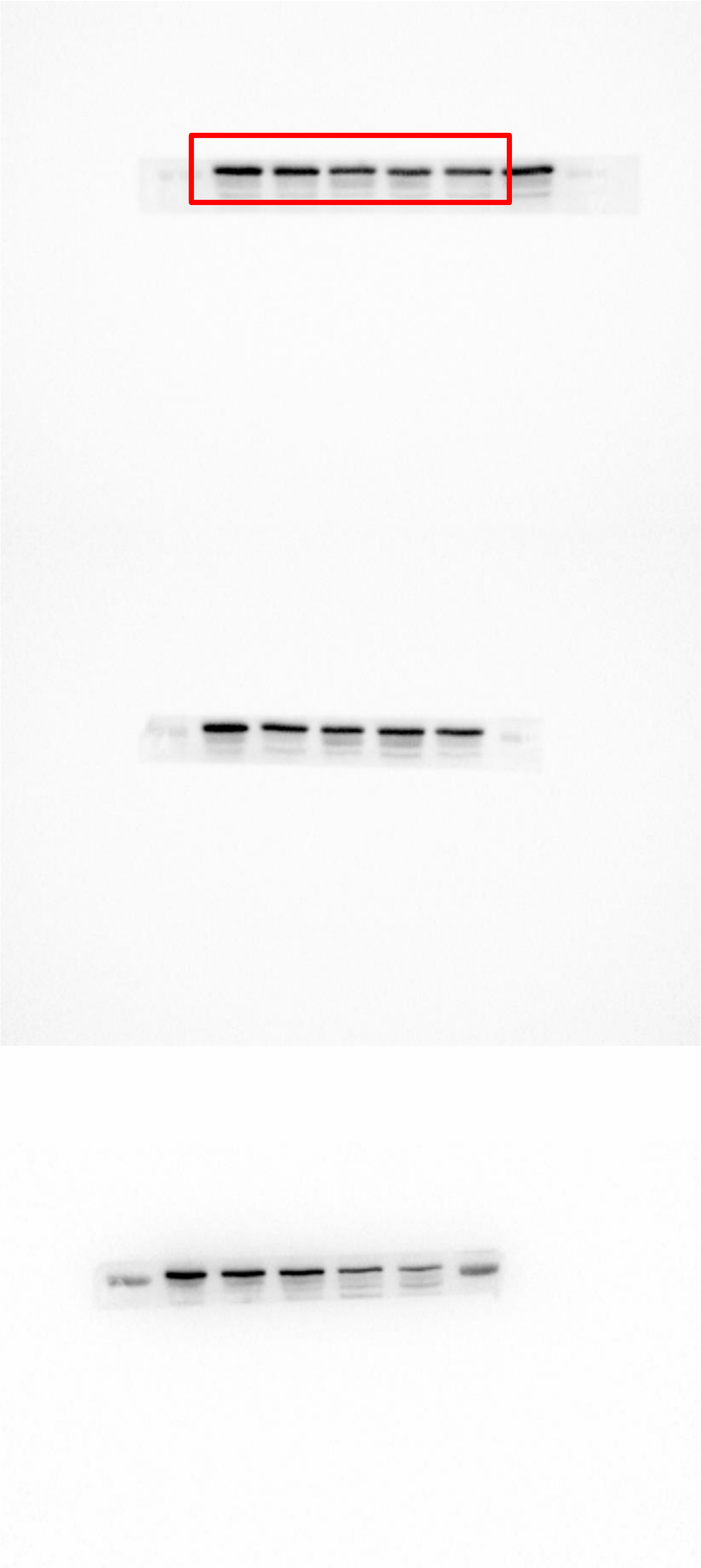

CDK2

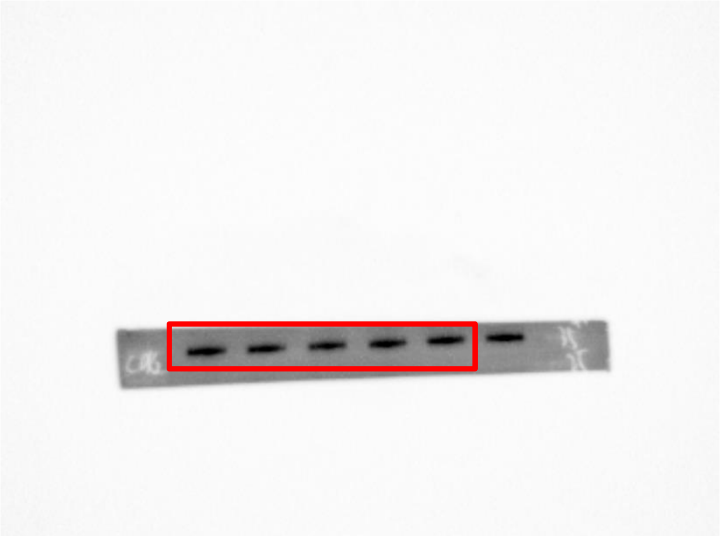

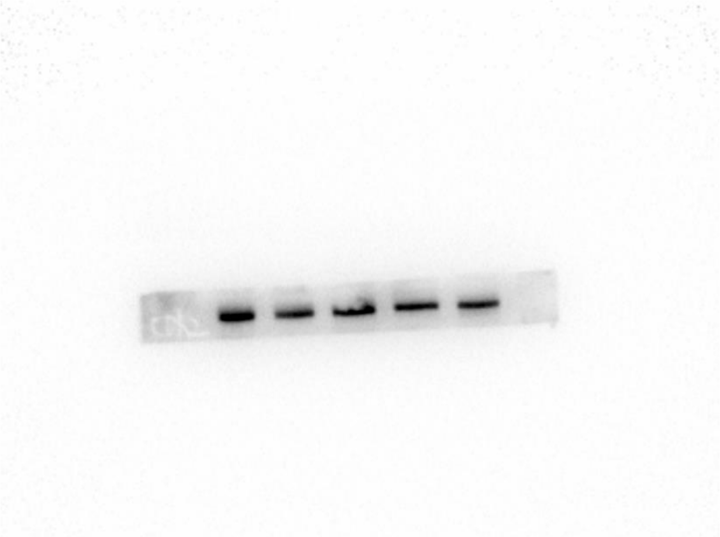

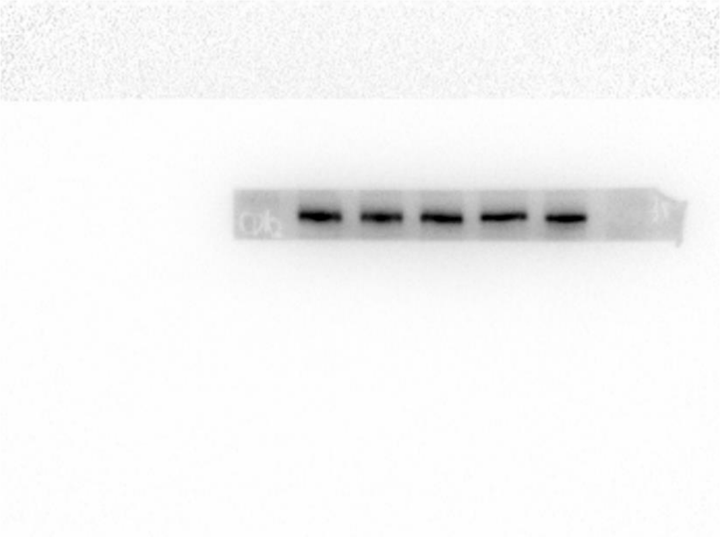

Cyclin D

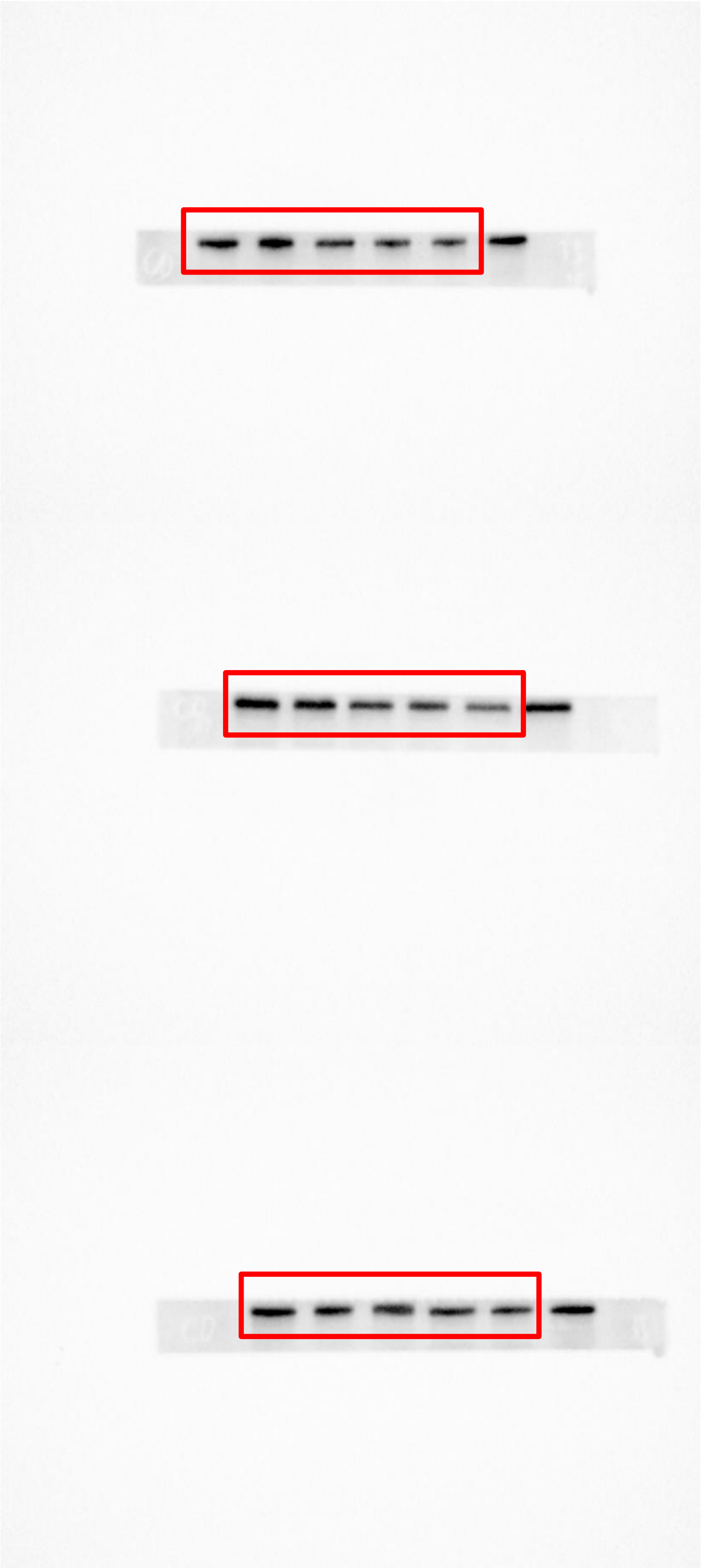

CDK4

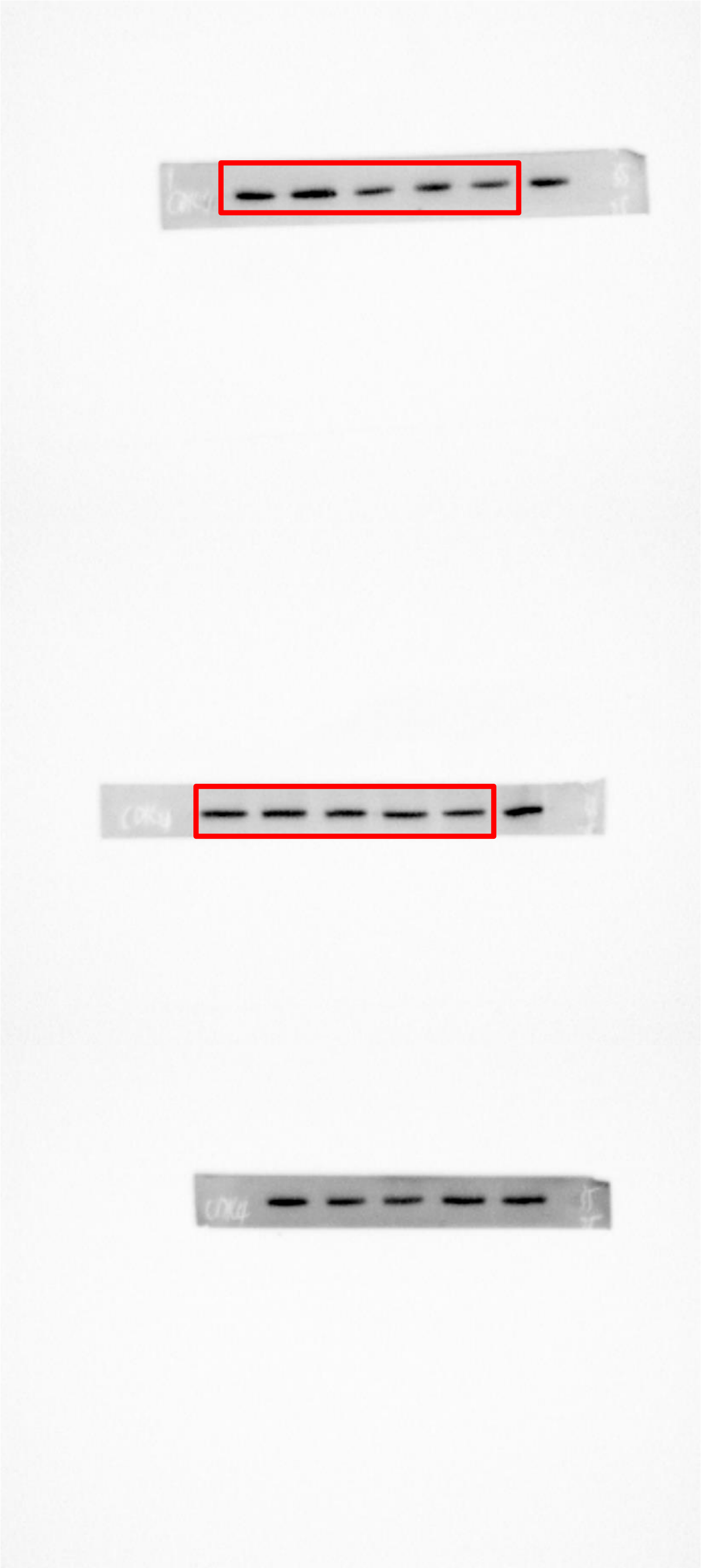

α-Tubulin

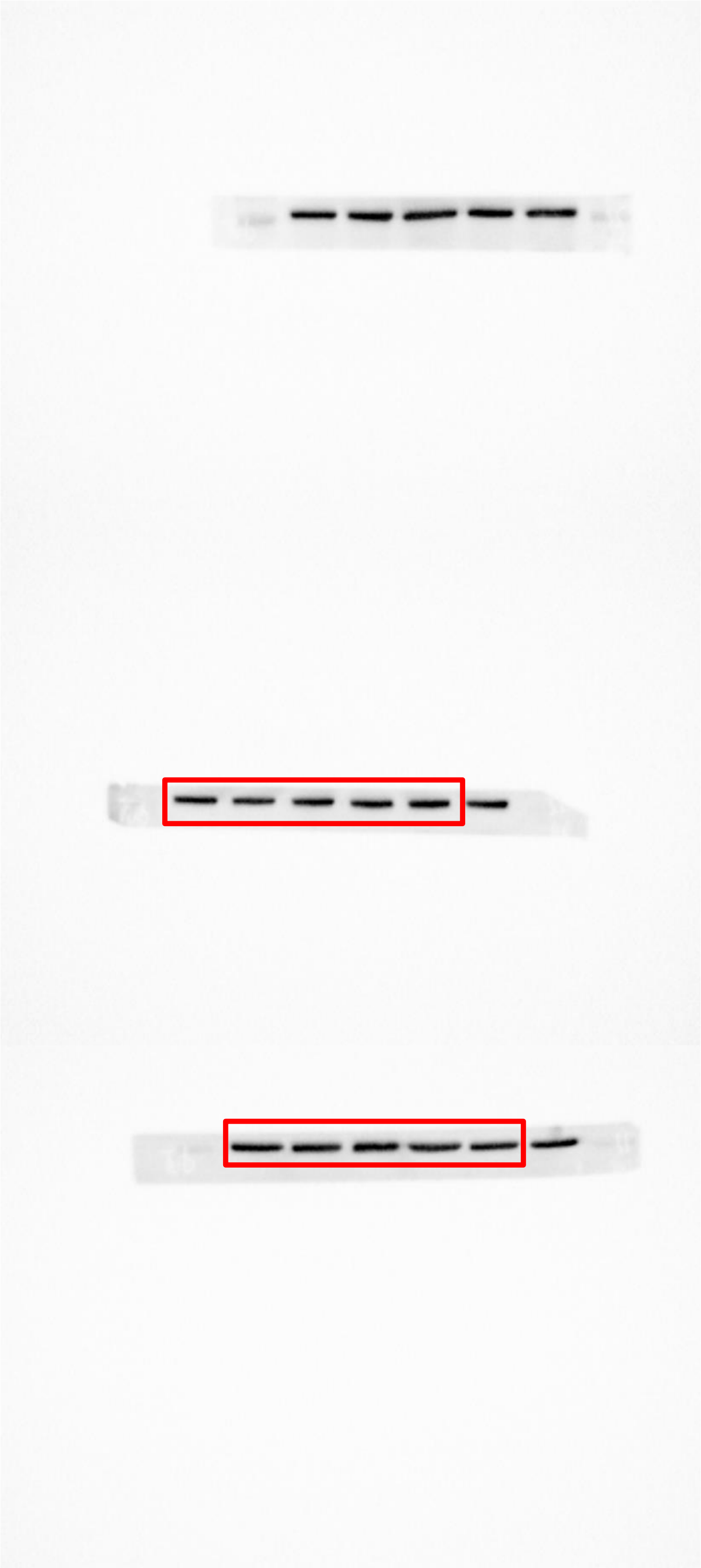

3.3 Uncropped Western Blots of A549 cells associated with apoptosis-regulating proteins

PARP1

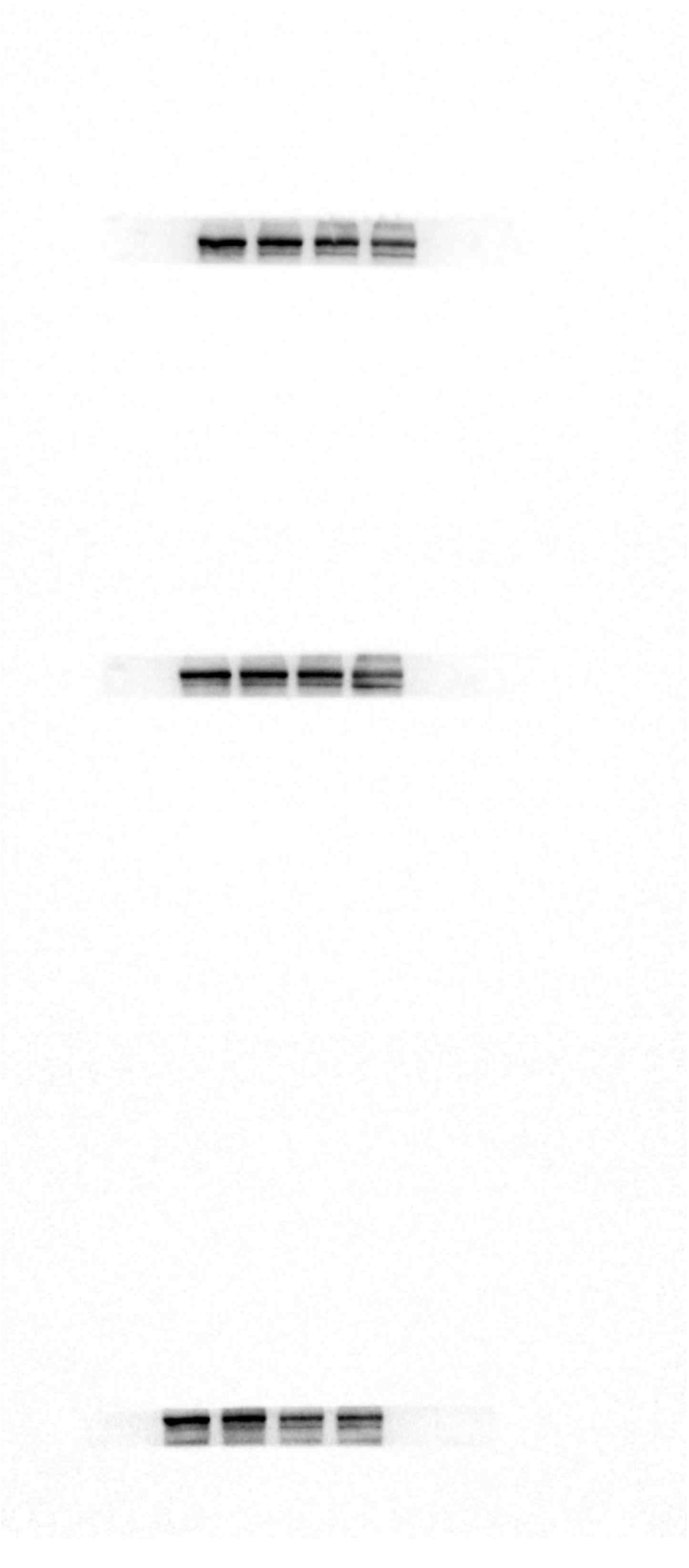

pro-Caspase 3

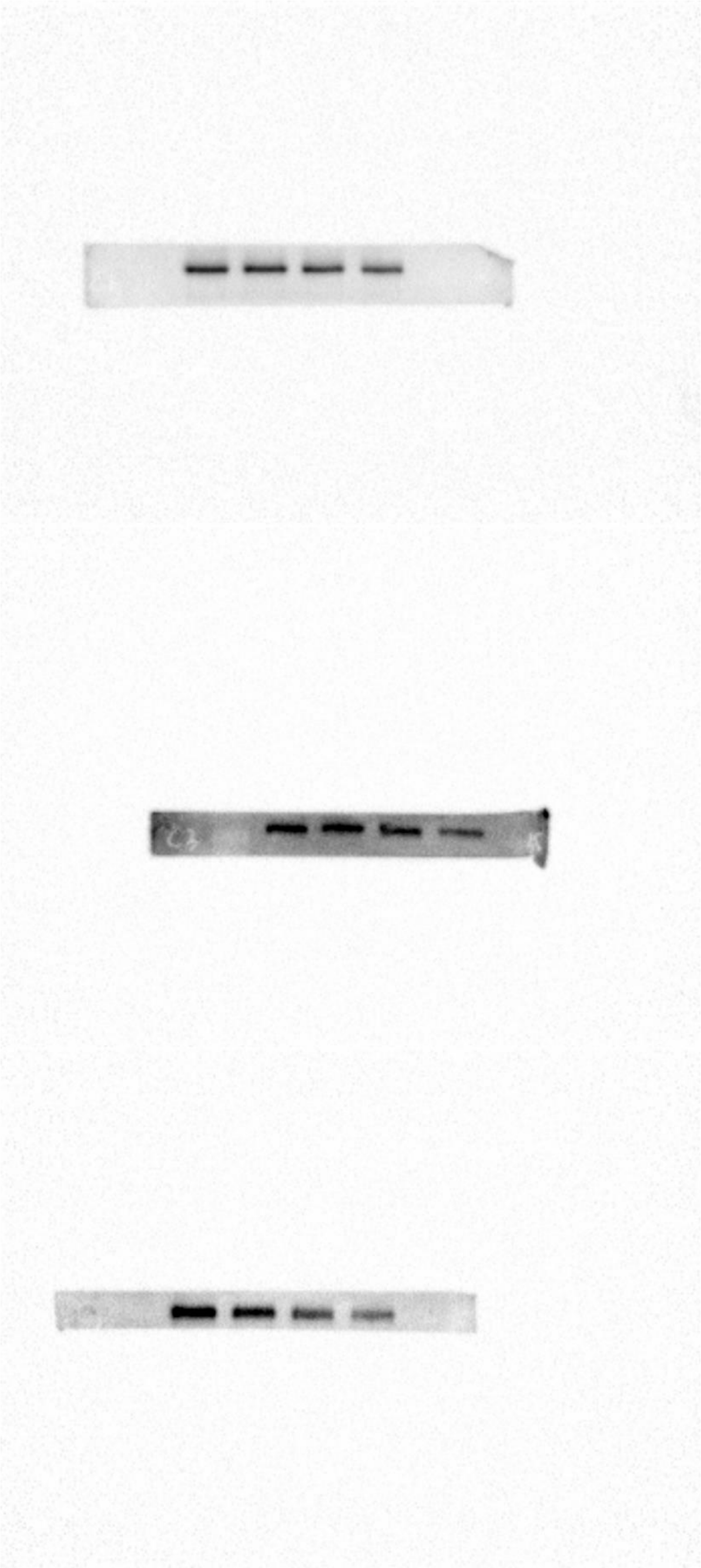

pro-Caspase 9

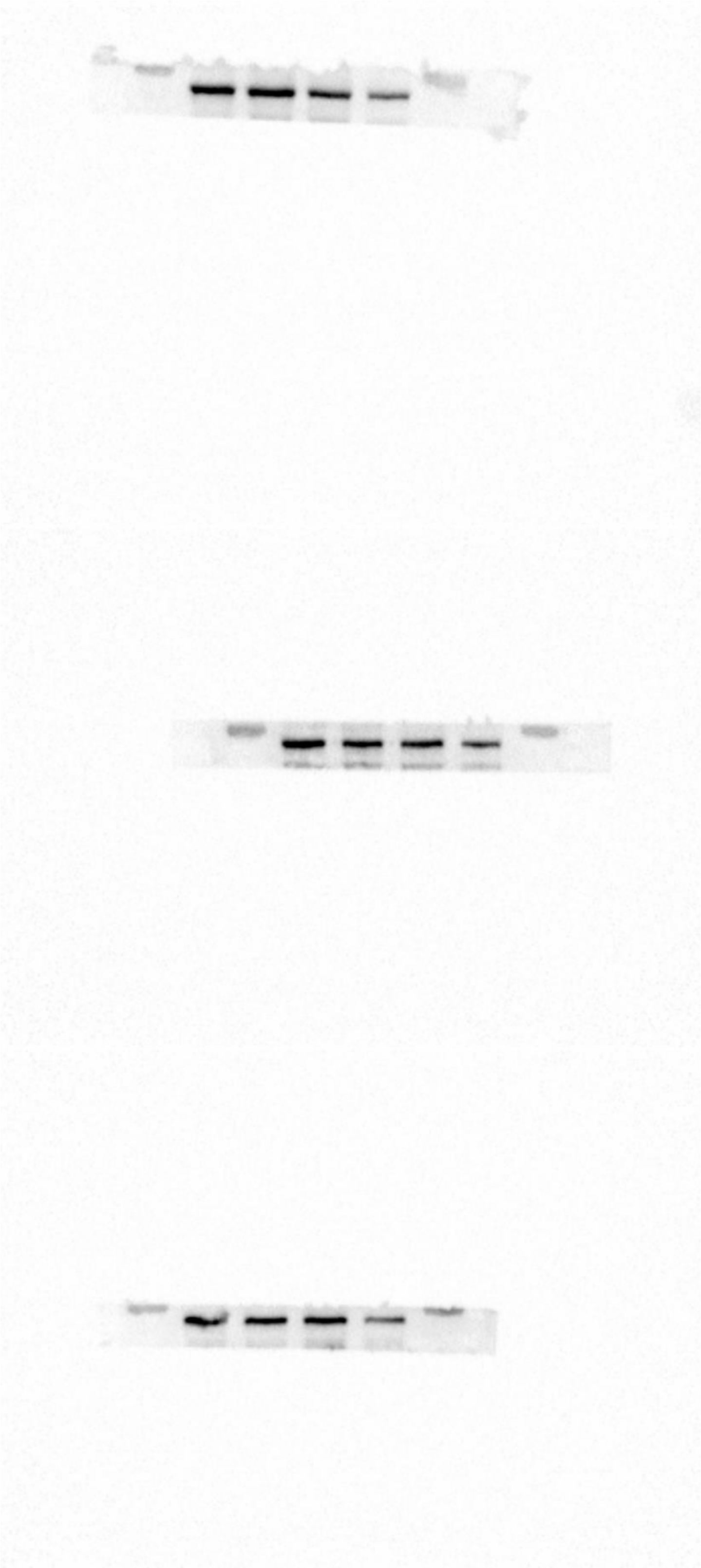

Bcl-2

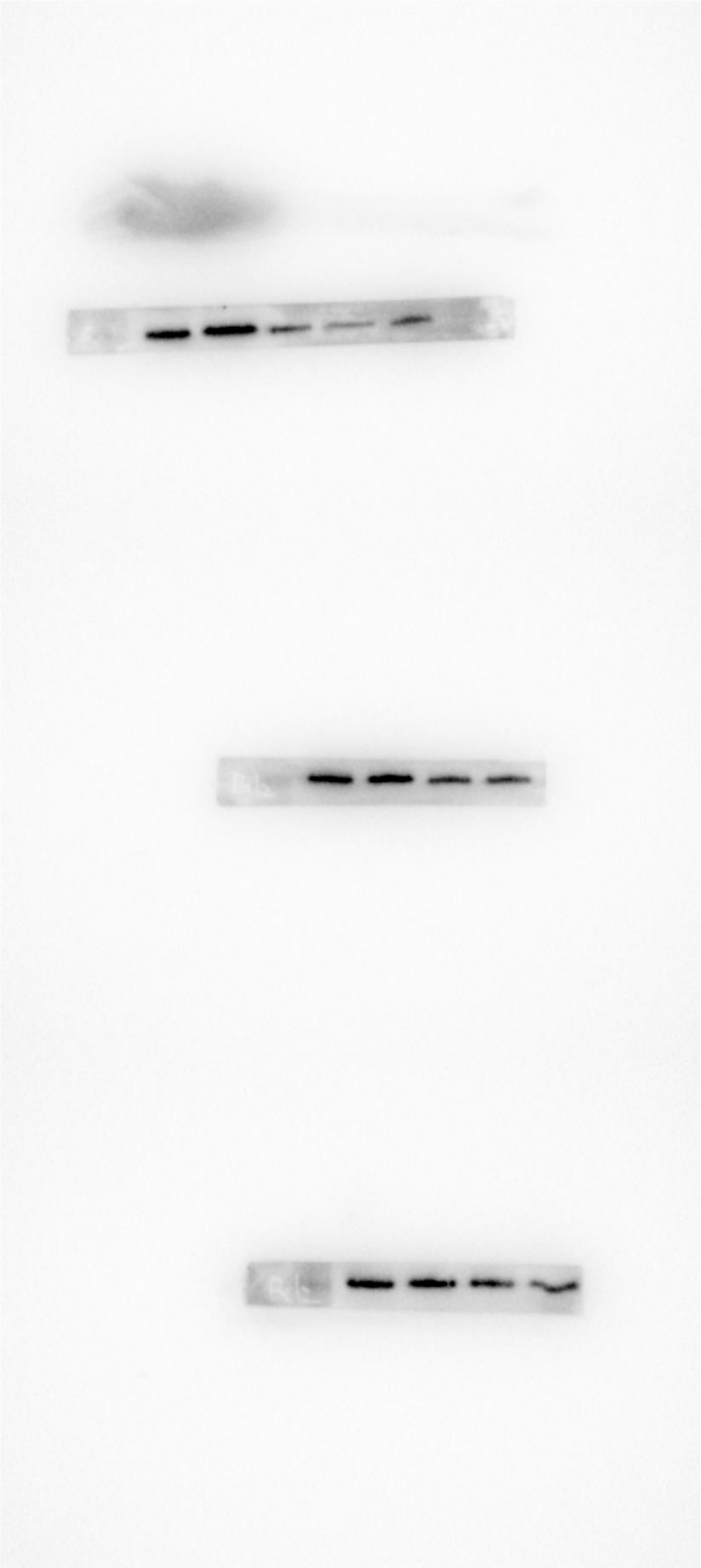

Bax

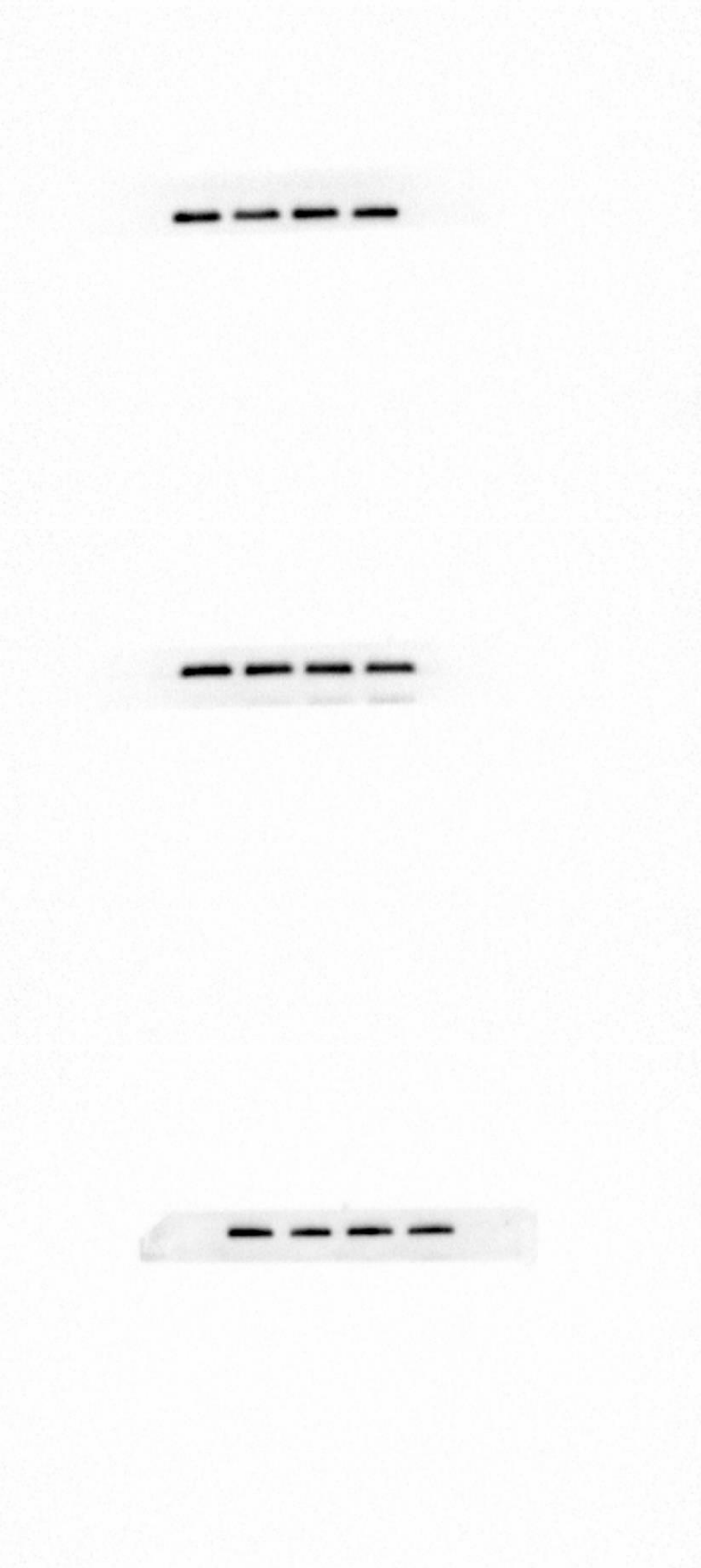

α-Tubulin

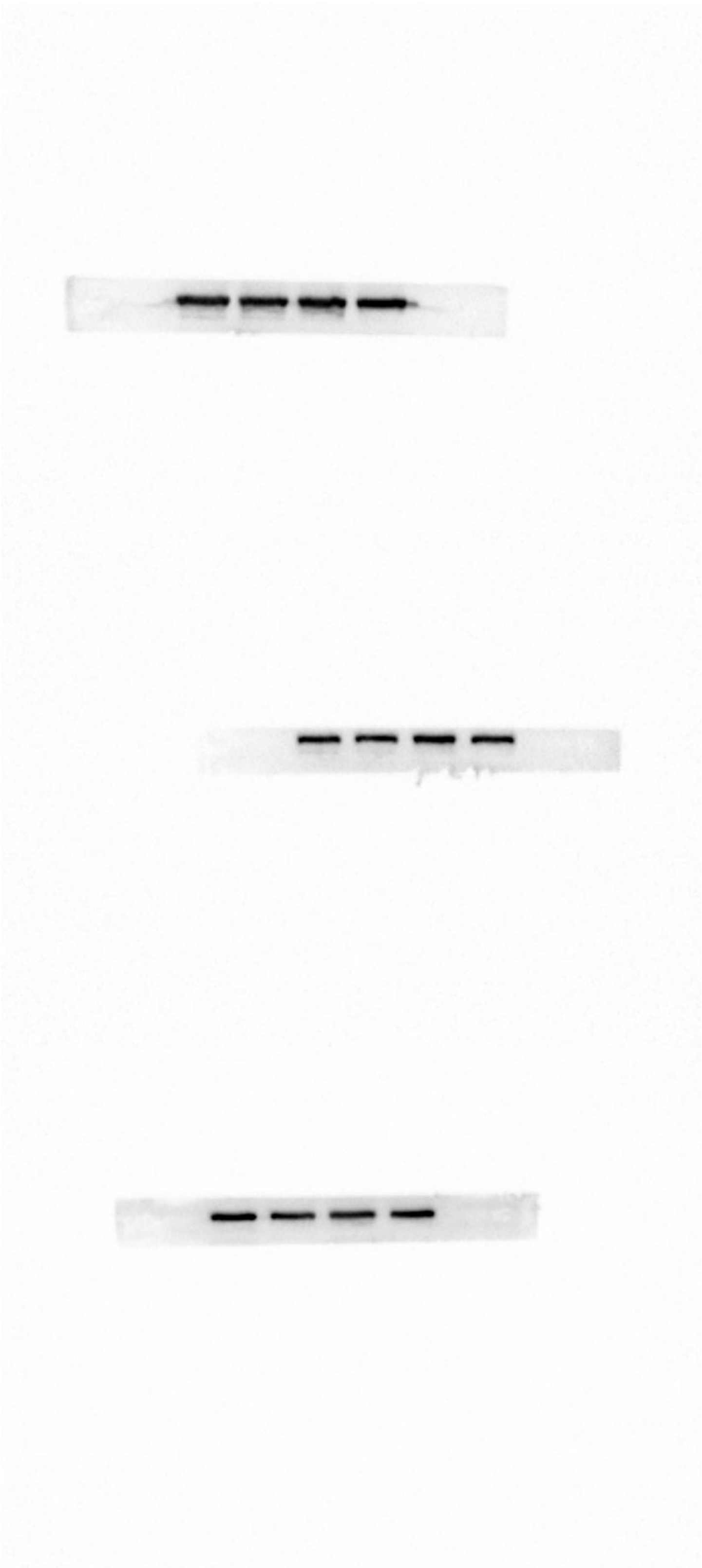

pro-Caspase 8

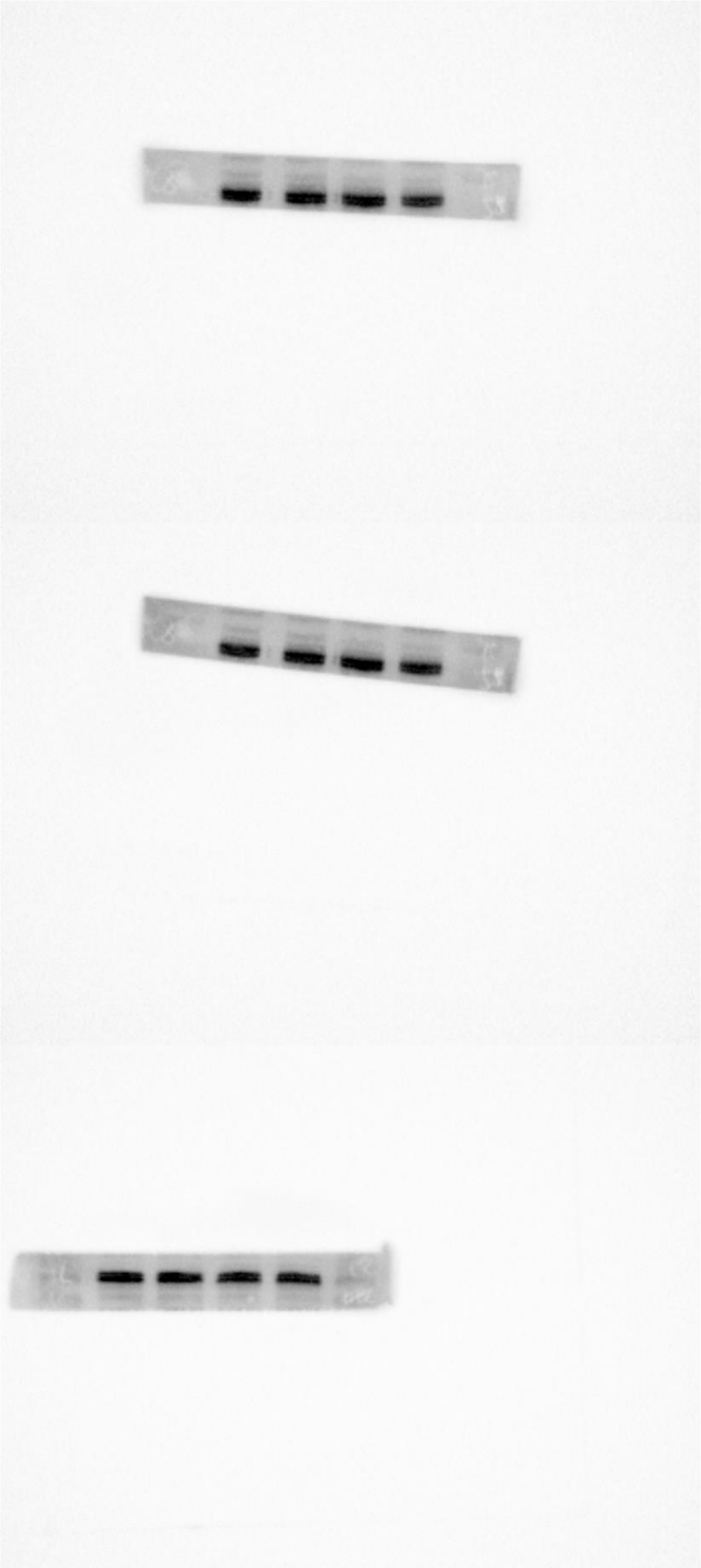

CHOP

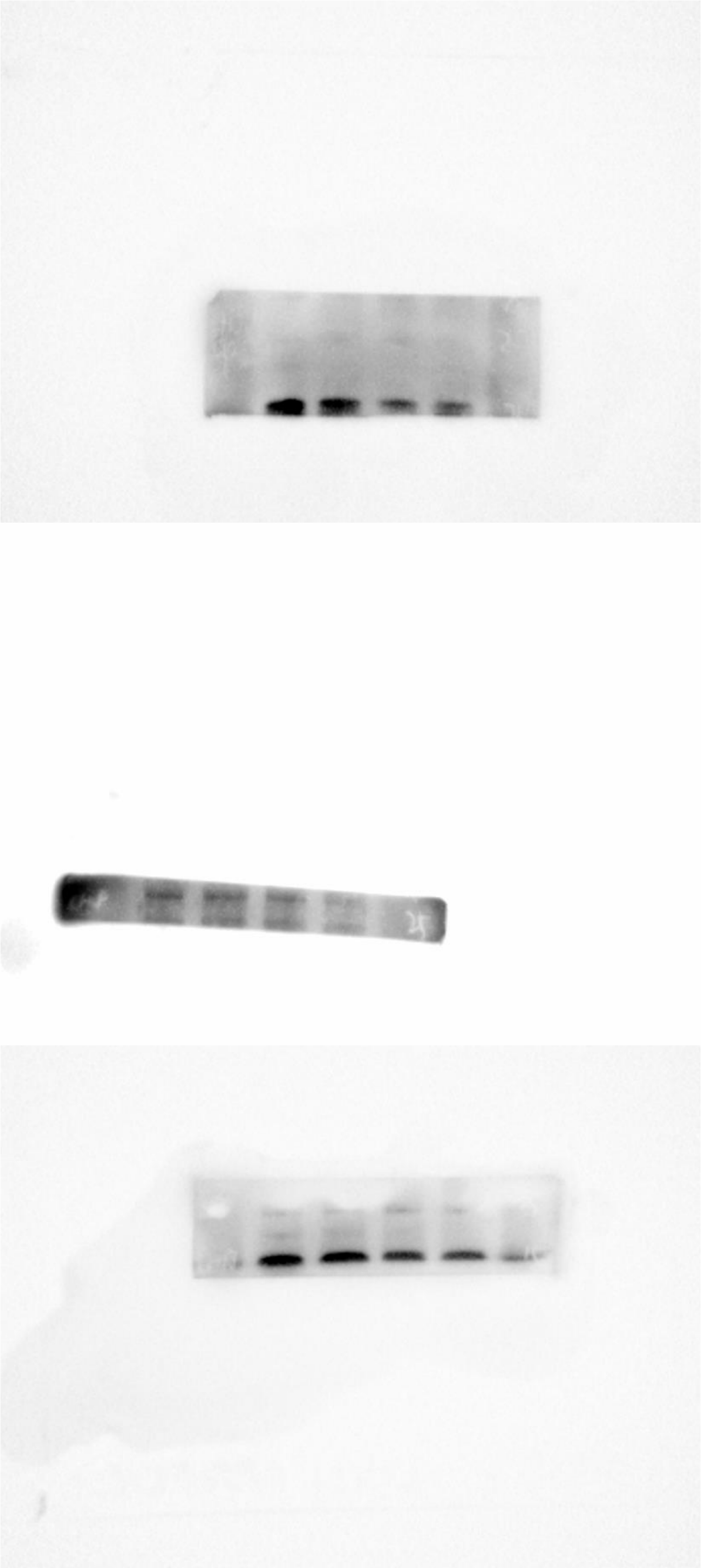

GRP78

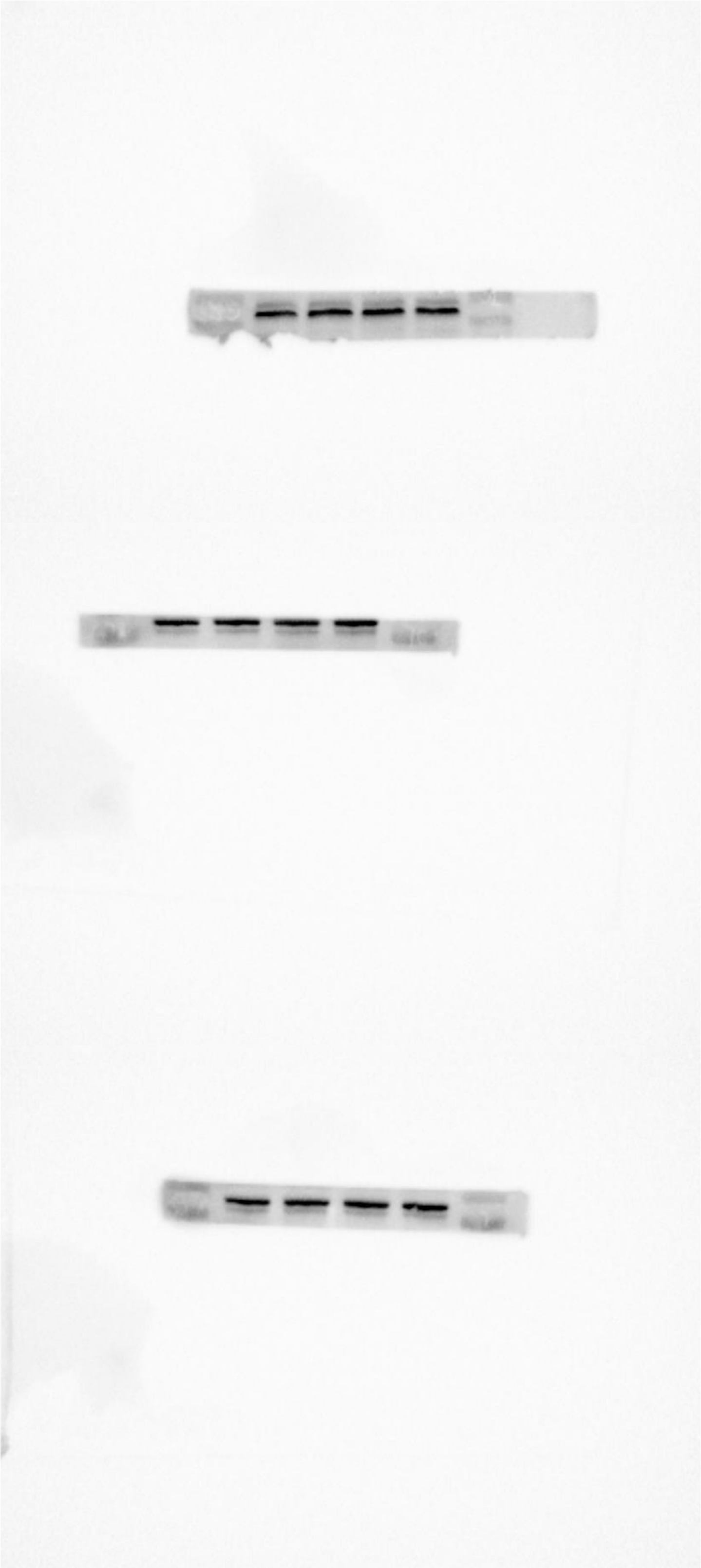

GAPDH

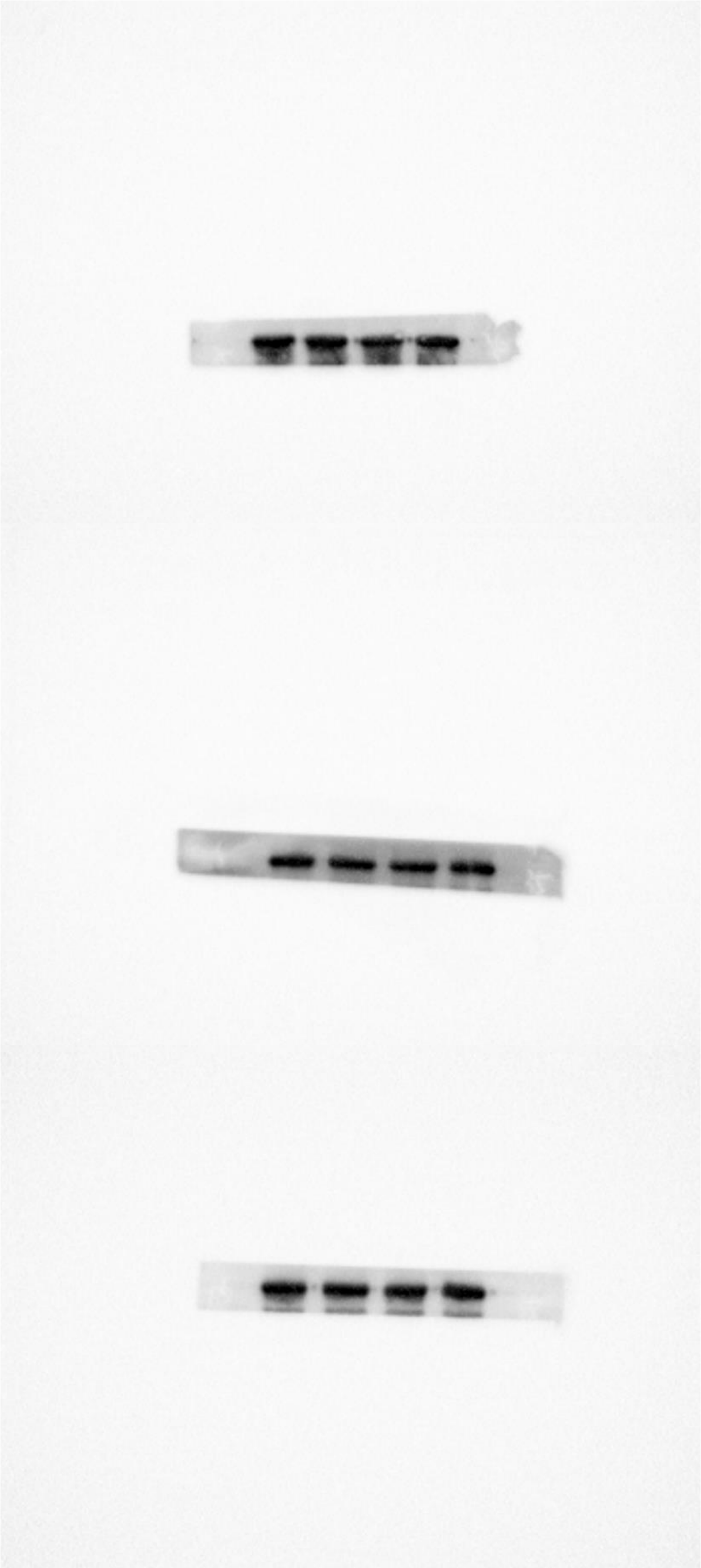

3.4 Uncropped Western Blots of proteins associated with the TNF signaling pathway in A549 cells

P65

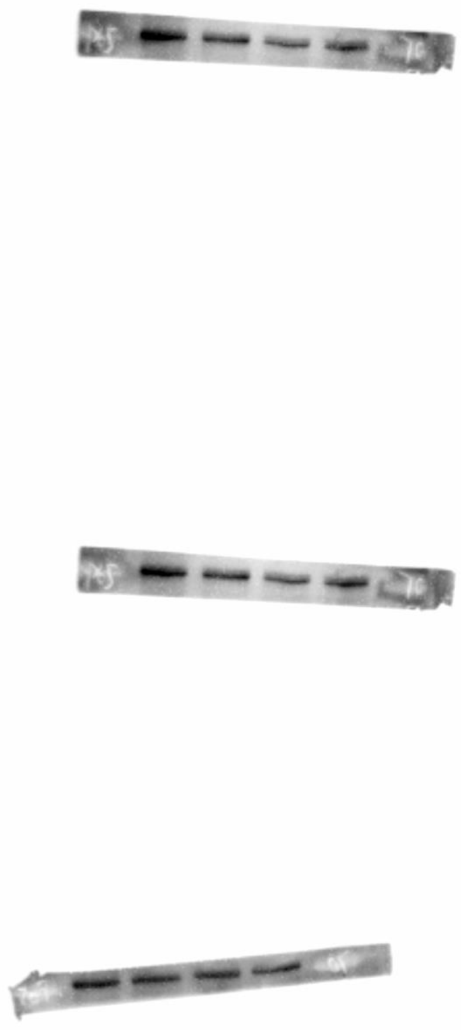

P50

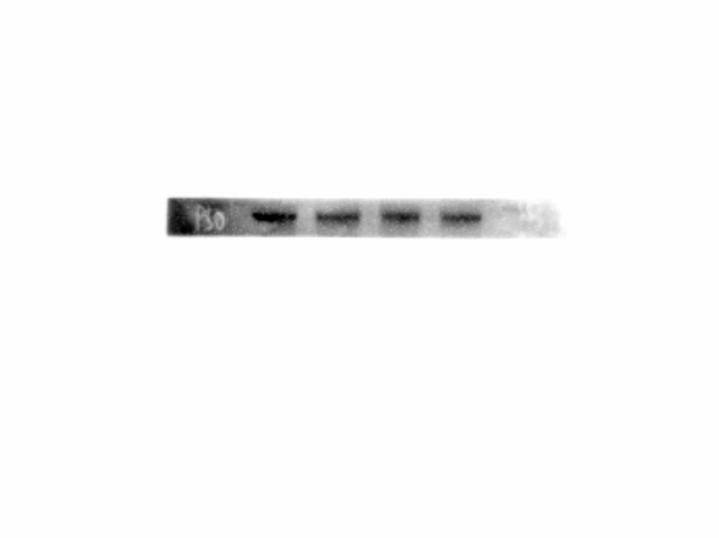

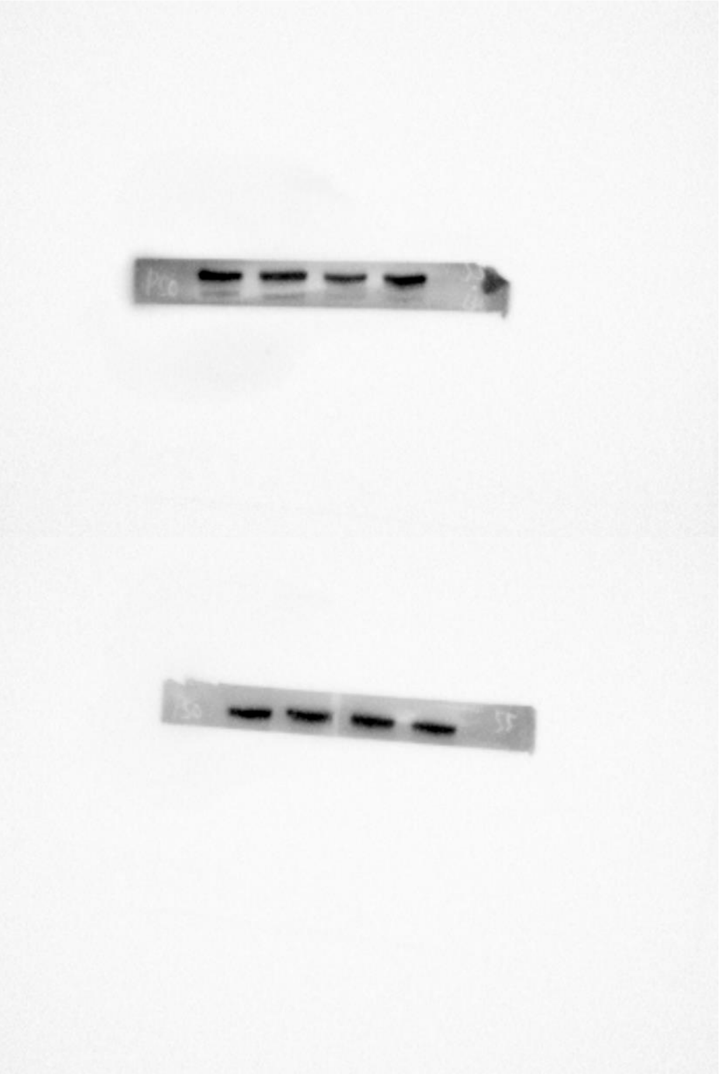

P105

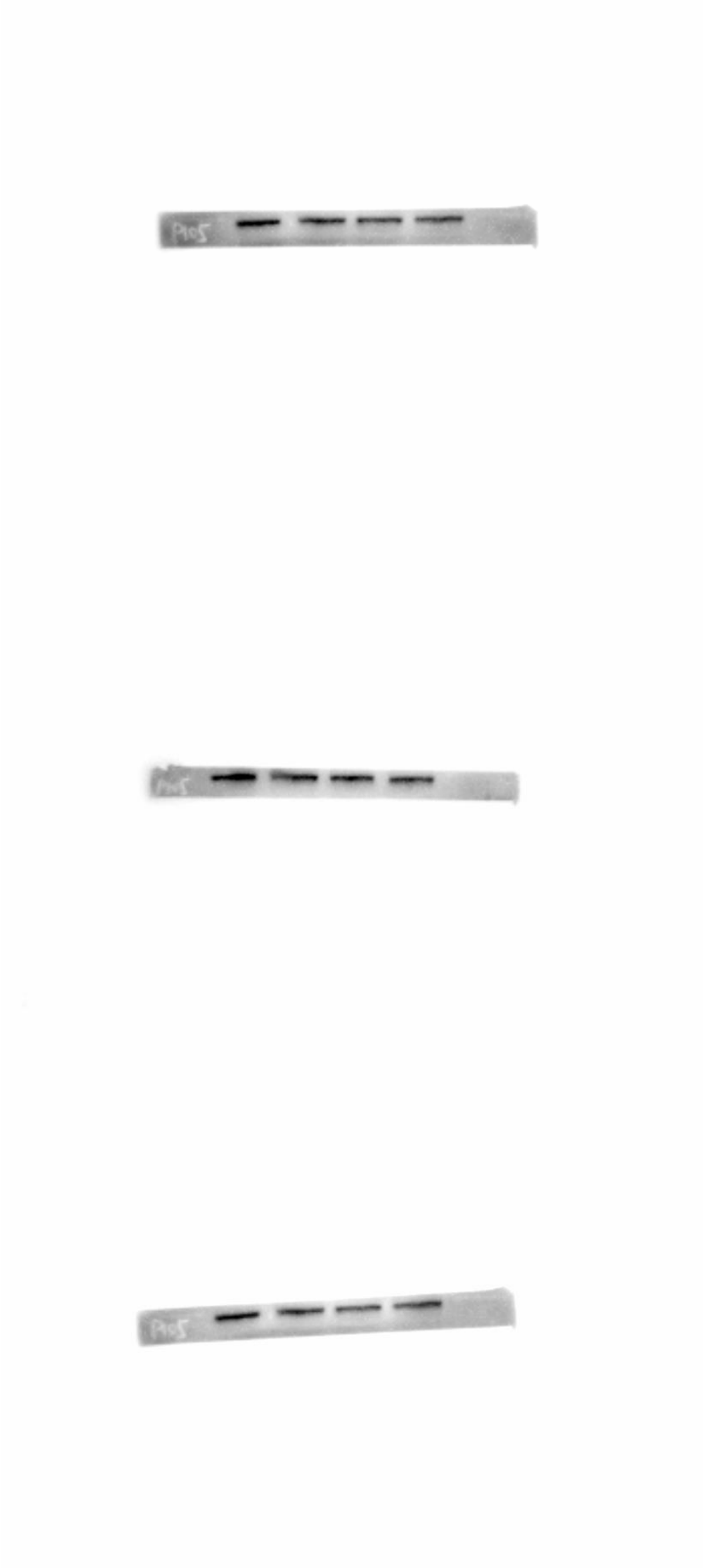

P38

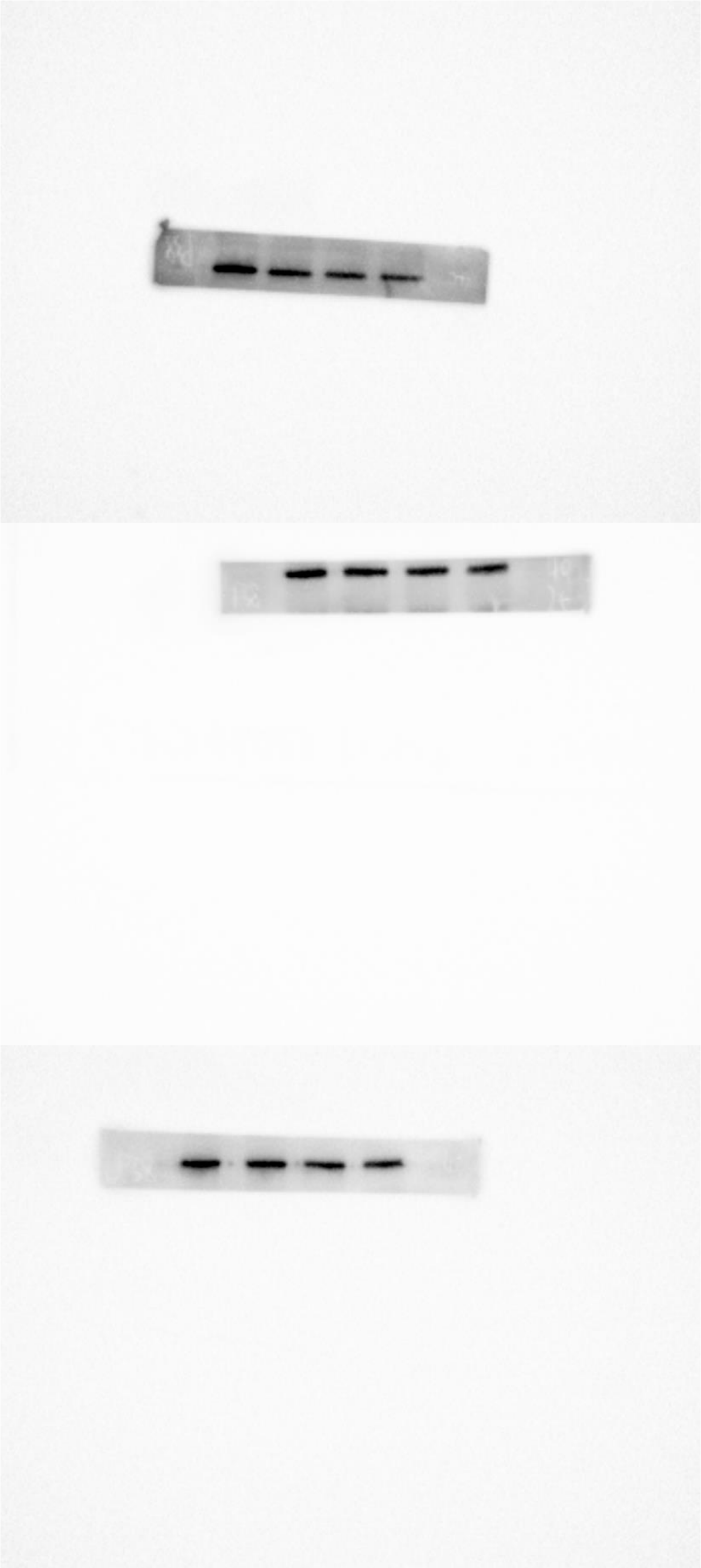

JNK

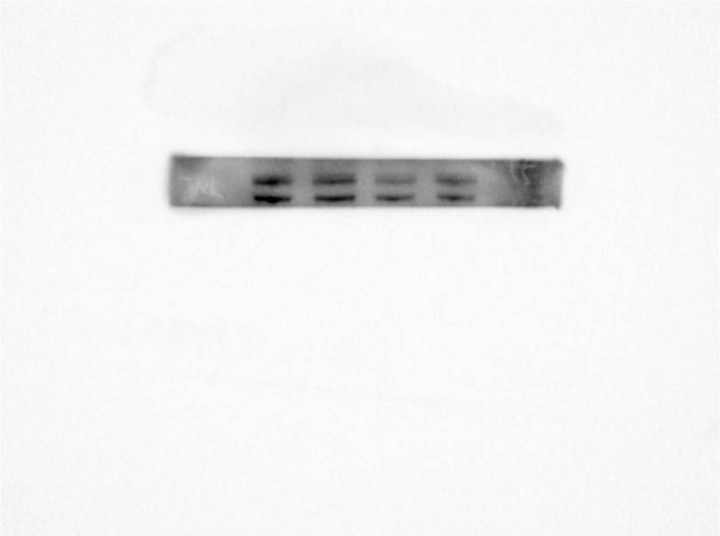

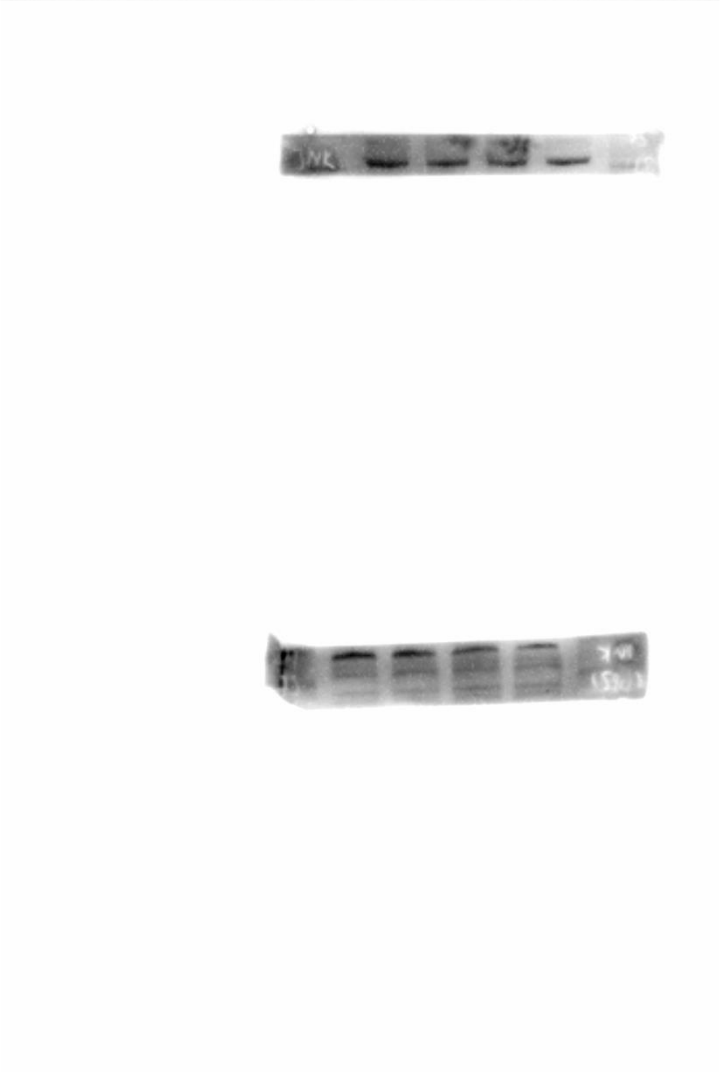

Supplement: Supplementary file 1 [file DataSheet1.pdf]
